# Power-efficient ultra-broadband soliton microcombs in resonantly-coupled microresonators

**DOI:** 10.1038/s41377-026-02186-9

**Published:** 2026-03-30

**Authors:** Kaixuan Zhu, Xinrui Luo, Yuanlei Wang, Ze Wang, Tianyu Xu, Du Qian, Yinke Cheng, Junqi Wang, Haoyang Luo, Yanwu Liu, Xing Jin, Zhenyu Xie, Xin Zhou, Min Wang, Jian-Fei Liu, Xuening Cao, Ting Wang, Shui-Jing Tang, Qihuang Gong, Bei-Bei Li, Qi-Fan Yang

**Affiliations:** 1 State Key Laboratory for Artificial Microstructure and Mesoscopic Physics and Frontiers Science Center for Nano-optoelectronics, School of Physics, Peking University, Beijing, China; 2 Beijing National Laboratory for Condensed Matter Physics, Institute of Physics, Chinese Academy of Sciences, Beijing, China; 3 National Biomedical Imaging Center, College of Future Technology, Peking University, Beijing, China; 4 Peking University Yangtze Delta Institute of Optoelectronics, Nantong, Jiangsu China; 5 Collaborative Innovation Center of Extreme Optics, Shanxi University, Taiyuan, China

**Keywords:** Solitons, Integrated optics, Frequency combs

## Abstract

The drive to miniaturize optical frequency combs for practical deployment has spotlighted microresonator solitons as a promising chip-scale candidate. However, these soliton microcombs could be very power-hungry when their span increases, especially with fine comb spacings. As a result, realizing an octave-spanning comb at microwave repetition rates for direct optical-microwave linkage is considered not possible for photonic integration due to the high power requirements. Here, we introduce the concept of resonant-coupling to soliton microcombs to reduce pump consumption significantly. Compared to conventional waveguide-coupled designs, we demonstrate (i) a threefold increase in spectral span for high-power combs and (ii) up to a tenfold reduction in repetition frequency for octave-spanning operation. This configuration is compatible with laser integration and yields reliable, turnkey soliton generation. By eliminating the long-standing pump-power bottleneck, microcombs will soon become readily available for portable optical clocks, massively parallel data links, and field-deployable spectrometers.

## Introduction

Two decades after their invention, optical frequency combs are coming out of laboratories to the real world^1–3^. Accelerating this trend demands further reductions in size and power consumption. Soliton microcombs offer a chip-scale solution: generated in high-Q nonlinear microresonators pumped by continuous-wave lasers, they exploit the balance between Kerr nonlinearity and anomalous dispersion to produce repetitive pulse trains, which manifest as phase-coherent teeth equally spaced by the repetition rate in the spectral domain^4–6^. These microcombs hold promise for on-chip optical frequency synthesizers^7^, clocks^8,9^, and spectrometers^10–14^, and their wide mode spacing (tens of gigahertz) suits wavelength-division multiplexing in communications^15–18^.

**Fig. 1:**
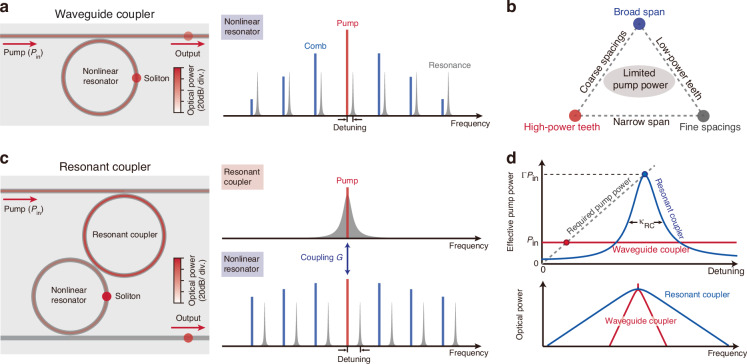
Pumping strategies of soliton microcombs. **a**, **c** Left: configurations of a nonlinear microresonator pumped via a waveguide coupler (**a**) or a resonant coupler (**c**), with the optical power indicated by color. Right: corresponding diagrams of energy flow. **b** The “impossible trinity” of soliton microcombs under limited pump power. **d** Top: effective pump power versus detuning. The dashed grey line denotes the minimum pump power required for soliton microcombs. Red and blue dots indicate the maximum detuning for soliton microcombs generated using waveguide couplers and resonant couplers, respectively. Bottom: optical spectra for soliton microcombs at the two detunings, obtained using waveguide couplers (red) and resonant couplers (blue)

Key performance metrics of any comb source are its span, power, and spacing. Fine spacing eases direct electrical detection; octave-spanning bandwidth enables carrier-envelope offset measurements through f-2f self-referencing^19^; high-power teeth maximize data throughput in communications. However, these metrics are usually coupled (Fig. 1a). In conventional soliton microcomb architecture, a nonlinear microresonator (NR) is evanescently coupled to a bus waveguide, in which four-wave mixing initiates when the input power ${P}_{{\rm{in}}}$ exceeds the threshold ${P}_{{\rm{th}}}$, but stable soliton formation further demands red-detuned pump and additional power. The 3-dB bandwidth $\varDelta {f}_{3{\rm{dB}}}$, central-tooth power ${P}_{{\rm{c}}}$, and repetition rate ${f}_{{\rm{r}}}$ are constrained by available pump power ${P}_{{\rm{in}}}$:

1 $$\frac{{P}_{{\rm{c}}}\varDelta {f}_{3{\rm{dB}}}^{2}}{{f}_{r}^{2}}\le 3.1\times {\eta }_{{\rm{NR}}}^{2}{P}_{{\rm{in}}}$$

where ${\eta }_{{\rm{NR}}}={\kappa }_{{\rm{e}},{\rm{NR}}}/{\kappa }_{{\rm{NR}}}$ is the loading factor of the NR, with ${\kappa }_{{\rm{NR}}}$ and ${\kappa }_{{\rm{e}},{\rm{NR}}}$ denoting the NR’s dissipation rate and the coupling rate to the waveguide, respectively (see Materials and methods). This “impossible trinity” cannot be simultaneously optimized given the limited pump power available from on-chip lasers (Fig. 1b). Also, the quadratic scaling law indicates that increasing the bandwidth or reducing the repetition rate is more challenging than increasing the tooth power.

Several strategies have been proposed to relax this constraint^20^. In particular, resonant couplers (RCs) – tested in fiber^21^ and electro-optic resonators^22^ – can enhance pump delivery and broaden comb spectra. Here, we demonstrate resonantly-coupled soliton microcombs, achieving up to threefold wider bandwidths and the first octave-spanning soliton combs at microwave repetition rates using only a continuous-wave pump.

## Results

### Principle

Our architecture, illustrated in Fig. 1c, interposes an auxiliary microresonator (RC) between the bus waveguide and the NR. In this configuration, the RC provides a resonant enhancement of the pump power, which is then delivered to the pump resonance of the NR via inter-resonator coupling. When the pump laser is tuned to the RC resonance, the effective pump power delivered to the NR is enhanced by a factor on the order of

2 $$\varGamma =\frac{4{G}^{2}}{{\kappa }_{{\rm{RC}}}{\kappa }_{{\rm{NR}}}}$$

where $G$ is the coupling rate between the resonators and ${\kappa }_{{\rm{RC}}}$ is the RC’s dissipation rate (see Supplementary Information). To suppress unwanted parametric oscillations in the RC, we typically set ${\kappa }_{{\rm{RC}}}\gg {\kappa }_{{\rm{NR}}}$. The enhancement can be considerable for $G\gg {\kappa }_{{\rm{NR}}},{\kappa }_{{\rm{RC}}}$, such that this resonant coupler can outperform a direct waveguide coupler over a bandwidth set by ${\kappa }_{{\rm{RC}}}$ (Fig. 1d). Since the maximum accessible detuning scales with pump power (dashed grey line in Fig. 1d), we can thus access much larger detunings once the RC resonance is red-detuned relative to the NR resonance. This also dramatically increases the soliton span (scales as $\sqrt{\delta \omega }$), which can also be inferred from Eq. 1 by applying the enhancement to the pump power.

### High-power ultra-broadband soliton microcombs

**Fig. 2:**
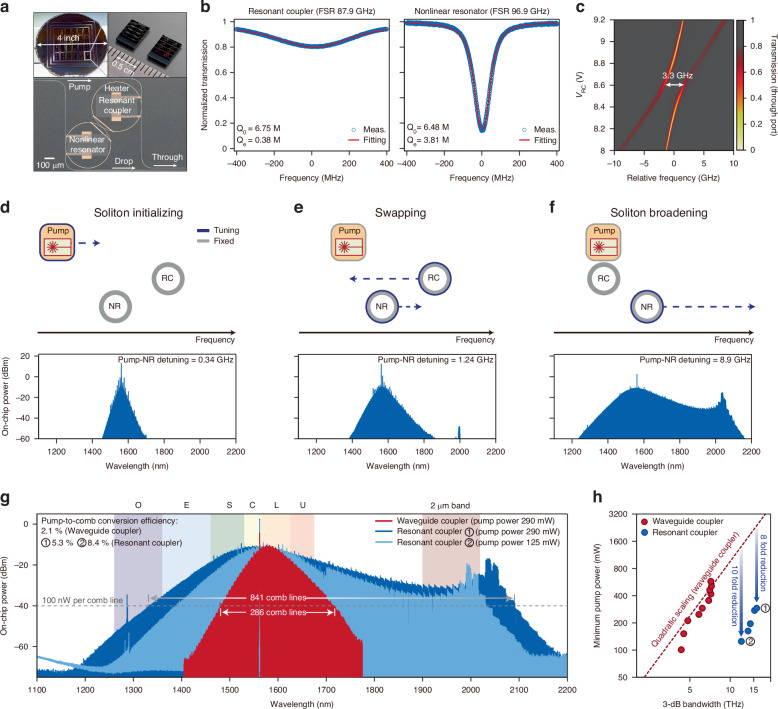
High-power ultra-broadband soliton microcombs. **a** Photos of the wafer, chips, and the coupled Si_3_N_4_ microresonators. **b** Measured transmission spectra revealing the intrinsic quality factor ${Q}_{0}$ and the external coupling quality factor ${Q}_{{\rm{e}}}$ for both the resonant coupler and the nonlinear microresonator. **c** Transmission spectra from the through port as a function of the voltage (*V*_RC_) applied to the resonant coupler’s heater. The minimum frequency difference between the hybridized modes is 3.3 GHz. **d–f** Sequential stages for generating ultra-broadband solitons in a resonantly-coupled NR. Top panel: the relative frequency positions and tuning directions of the pump, RC, and NR. Bottom panel: corresponding optical spectra of soliton microcombs. **g** Comparison of optical spectra for soliton microcombs generated using conventional waveguide couplers (red) and resonant couplers (dark and light blue). All power refers to on-chip power. The pump powers on the bus waveguide, and the pump-to-comb conversion efficiencies are indicated. Communication bands covered by optical amplifiers are highlighted with different color shadings. **h** Measured minimum pump power as a function of 3 dB bandwidth of soliton microcombs pumped via the waveguide coupler (red dots) and the resonant coupler (blue dots) on a log-log scale. The red dashed line represents the quadratic scaling

We implement our design in 786 nm-thick Si_3_N_4_ microresonators fabricated via subtractive processing (see Materials and methods). The RC (waveguide width 1.5 μm) and NR (1.8 μm) have free-spectral ranges (FSRs) of 87.9 GHz and 96.9 GHz, respectively (Fig. 2a). Both resonators exhibit intrinsic ${Q}_{0}\sim 7\times {10}^{6}$; the RC is overcoupled (${Q}_{{\rm{e}}}\approx 0.38\times {10}^{6}$), while the NR has ${Q}_{{\rm{e}}}\approx 3.8\times {10}^{6}$ (Fig. 2b). Integrated heaters provide tuning of the resonances. Adjusting the heater power allows for observation of avoided crossings between two resonances, which reveals inter-resonator coupling $G/2\pi =1.65$ GHz (Fig. 2c) and predicts $\varGamma \sim 100$.

Soliton initiation via the RC differs from conventional schemes (see Supplementary Information). With the RC initially blue-detuned, we sweep the pump into the NR resonance to access single solitons in $\sim 80 \%$ of trials (see Figure S6 in Supplementary Information). At 0.34 GHz detuning, the comb spans 234 nm at −60 dBm (Fig. 2d), but exhibits spectral spurs arising from avoided mode crossings between multiple pairs of NR and RC resonances across the spectrum. Tuning the RC red and NR blue leads to the swapping of their frequencies, which also increases detuning to 1.24 GHz and broadens the comb span to 488 nm (Fig. 2e). At the final stage, we red-shift the NR to 9 GHz detuning to extend the comb span to 937 nm (Fig. 2f). At such large detuning, the NR spectrum becomes smooth because avoided mode crossings in the NR dispersion are comparably negligible (see Figure S2 in Supplementary Information). In this regime, the pump resonances of the RC and NR are only weakly hybridized owing to their frequency separation, in contrast to other efficient-pumping strategies that require alignment of the two resonances^23^. Further increasing the detuning would cause modulational instability in the RC, which would destabilize the soliton (see Supplementary Information). This reduces the maximum detuning from the theoretical prediction, and could be overcome by further reducing the $Q$ of the RC.

Owing to the NR’s large group-velocity dispersion (see Figure. S4b in Supplementary Information) and strong coupling to the bus waveguide, the comb attains -10.9 dBm tooth power at the center and covers O-band to 2 m (Fig. 2g). At 290 mW pump, we record 15.4 mW of total drop-port power (excluding the pump line), corresponding to 5.3% conversion efficiency, with an additional 54 mW of comb power emitted from the through port due to RC-NR leakage. At a lower pump power (125 mW), the spectrum remains broad, delivering a comb power of 10.46 mW, corresponding to 8.3% conversion efficiency.

Benchmarking against a waveguide-coupled NR (with identical geometry and consistent *Q*-factor) shows that at 290 mW pump, the conventional device achieves 6.2 THz 3 dB bandwidth with 286 lines over 100 nW, whereas the device using RC achieves 15.8 THz 3 dB bandwidth with 841 lines over 100 nW (Fig. 2h). Even with 600 mW launched into the bus, the waveguide-coupled device caps at 7.2 THz; extrapolating the quadratic pump-span scaling suggests more than 1.5 W and 2 W would be needed to match the RC’s performance at 125 and 290 mW pump power, respectively, which underscores up to 10-fold pump power enhancement afforded by resonant coupling (Fig. 2h). This enhancement factor represents a conservative estimate, since the pump power required for the waveguide-coupled device to achieve broader 3 dB bandwidths could be substantially higher than predicted by the quadratic scaling because of the soliton self-frequency shift induced by Raman effect^24,25^ and higher-order dispersion^26,27^.

### Octave-spanning soliton microcombs at microwave and millimeter-wave rates

**Fig. 3:**
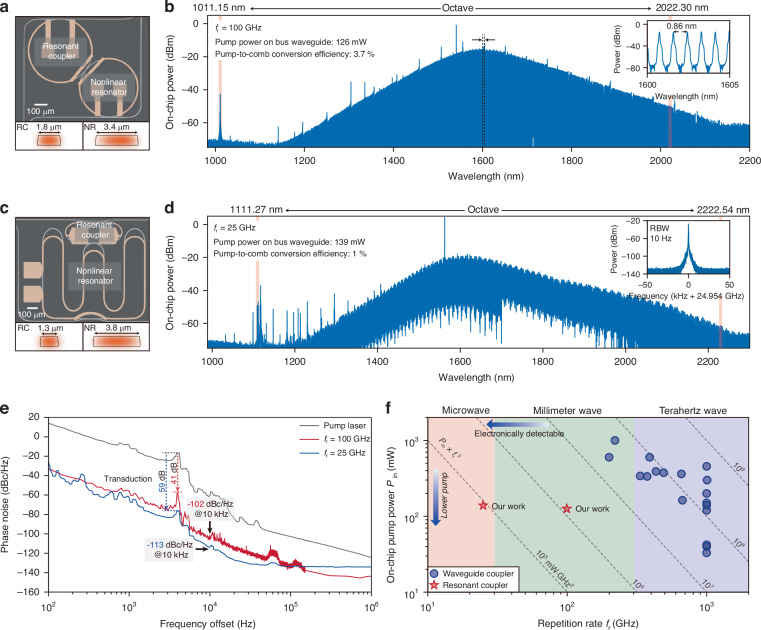
Octave-spanning soliton microcombs at millimeter wave and microwave rates. **a** Top: image of the coupled ring microresonators. Bottom: the cross-sectional profile of the TE fundamental mode. **b** Optical spectrum of the octave-spanning soliton microcomb at ${f}_{{\rm{r}}}$ of 100 GHz. Insets: zoom-in view of the spectrum between 1600 nm and 1605 nm. **c** Top: image of the coupled finger-shaped and racetrack microresonators. Bottom: the cross-sectional profile of the TE fundamental mode. **d** Optical spectrum of the octave-spanning soliton microcomb at ${f}_{{\rm{r}}}$ of 25 GHz. Inset: the electrical beat note at 24.954 GHz. RBW: resolution bandwidth. **e** Phase noise of the pump laser (grey) and the repetition rate of 100 GHz (red) and 25 GHz (blue) soliton microcombs, with noise transduction factor indicated. **f** Comparison of the on-chip pump powers and repetition rates of reported octave-spanning soliton microcombs pumped by continuous-wave lasers. Data from waveguide-coupled configurations are compiled from refs. ^42–45,49–59^

By widening the NR waveguide (Fig. 3a, c), we reduce its group-velocity dispersion that facilitates a broader comb span (see Figure. S4b, c in Supplementary Information). In a 100 GHz FSR device (3.4 μm width), 126 mW pump at 1541 nm delivers an octave spectrum from 1007 to 2130 nm (Fig. 3b), with coherent dispersive waves near 1011 nm confirmed by heterodyning against another laser (see Supplementary Information). In a 25 GHz device (finger-shaped NR and racetrack RC, 1 ${\mathrm{mm}}^{2}$ footprint), 139 mW pump at 1562 nm generates an octave spectrum from 1098 to 2250 nm (Fig. 3d). Direct photodetection of the soliton microcomb produces a monotone electrical beatnote that corresponds to the repetition rate. The spectral peaks observed in the 1100–1300 nm range mainly originate from dispersive waves induced by higher-order dispersion and avoided mode crossings, which are well reproduced by numerical simulations (see Supplementary Information). Both spectra exhibit notable shifts of the spectral-envelope center from the pump wavelength, which is driven by Raman self-frequency shifts^24,25,28^ and dispersive-wave recoil^6,29^. This limits the maximum comb span and must be balanced to realize even broader combs^25^.

To quantify coherence for future self-referencing, we measure the phase noise of the 100 GHz comb using a multi-frequency delayed self-heterodyne interferometer, and that of the 25 GHz comb with a commercial phase-noise analyzer (see Materials and methods). At a 10 kHz offset, we record −102 dBc/Hz (100 GHz) and –113 dBc/Hz (25 GHz), comparable to the lowest reported for free-running integrated soliton microcombs^30^ (Fig. 3e). To identify the dominant noise contribution, the phase noise of the pump laser is also characterized using the same delayed self-heterodyne interferometer. At low offset frequencies, the phase noise of the repetition rate and the pump exhibits similar spectral features, revealing direct noise transduction from the pump to the comb. The transduction factor, inferred from the peaks near 4 kHz offset, is approximately 41 dB for the 100 GHz comb and 59 dB for the 25 GHz comb.

Figure 3f plots the on-chip pump power required for octave-spanning comb generation versus repetition rate for various CW-pumped platforms. Because $${P}_{{\rm{in}}}\propto {f}_{{\rm{r}}}^{-2}$$ (Eq. 1), we compare the figure of merit $${P}_{{\rm{in}}}\times {f}_{{\rm{r}}}^{2}$$. Owing to the resonantly enhanced pump power, octave-spanning microcombs with substantially low repetition rates can be generated using low pump powers. Consequently, our RC architecture achieves values around $${10}^{5}$$ mW$$\cdot$$$${\mathrm{GHz}}^{2}$$, which is lower than the best results reported in conventional waveguide-coupled configurations by two orders of magnitude.

### Hybrid-integrated turnkey soliton microcombs

**Fig. 4:**
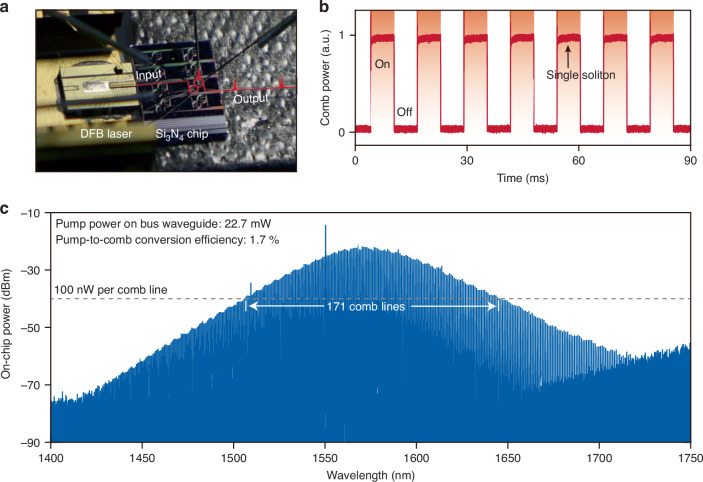
Hybrid-integrated turnkey soliton microcombs. **a** Photo showing a DFB laser directly coupled to a Si_3_N_4_ chip. **b** Measured comb power when the soliton is turned on and off 7 consecutive times. **c** Optical spectrum of a soliton microcomb

We use an on-chip laser to drive the soliton microcomb through an RC. A distributed-feedback (DFB) laser is coupled into the $${\mathrm{Si}}_{3}$$$$\mathrm{N}_{4}$$ chip, delivering approximately 20 mW of optical power to the bus waveguide (Fig. 4a). To enable operation at this low pump power, an NR with a higher $$Q$$ is selected (see Materials and methods). Without an optical isolator, light backscattered from the microresonator re-enters the laser cavity and perturbs its tuning. This phenomenon, known as self-injection locking^31–34^, narrows the laser’s linewidth and biases the system toward soliton microcomb generation when the feedback phase is appropriately tuned. In our implementation, the reinjection feedback phase is adjusted using a piezoelectric stage to meet the condition for stable soliton formation.

By optimizing the feedback phase, single-soliton microcombs emerge deterministically each time the laser current is tuned to a predetermined setpoint. To emulate soliton turn-on dynamics, the laser current is modulated with a square wave (Fig. 4b). Each time the current is switched to the target value, a single-soliton state reliably forms in the NR. Ultimately, this self-injection-locked pumping approach obtains single-soliton microcombs with a ${f}_{{\rm{r}}}$ of 99 GHz and an optical bandwidth exceeding 300 nm (Fig. 4c). 171 comb lines are above 100 nW. From the optical spectrum, the detuning is inferred to be approximately 0.72 GHz, corresponding to a pulse duration of about 33 fs. This is the broadest soliton microcombs at such a repetition rate when pumped by on-chip lasers^35^.

## Discussion

Generating octave-spanning microcombs at electronically accessible rates with low pump power is a milestone and can unlock many opportunities. First, optical frequency division^36^, optical frequency synthesis, and optical clocking based on self-referenced optical frequency combs can be implemented on chip without complex protocols involving multiple combs^7,8^. Second, these combs provide precise wavelength calibrators for astronomical spectrographs, delivering nearly 1600 comb lines within a 20 dB power variation—surpassing prior microcomb-based astrocombs and supporting radial-velocity precisions better than 25 cm/s (refs. ^37,38^). Third, in the time domain, they produce sub-20 fs pulses, which can be used for synthesizing low-duty-cycle femtosecond pulse trains and arbitrary optical waveforms. In addition, the 100-GHz-rate microcomb demonstrated in Fig. 2g is ideal for high-capacity wavelength-division-multiplexed optical communications, exhibiting only 1.7 dB variation in comb-tooth power across the C-band and more than 160 lines within a 3 dB bandwidth; without additional spectral flattening, it could in principle support aggregate data rates up to 64 Tb/s, assuming 400 Gb/s per channel^39^. These functionalities have been challenging to realize, or have suffered from limited performance, when using narrow-band microcombs.

The present microresonator design can be further improved. Parametric oscillations in the RC must be suppressed, for example by narrowing the RC waveguide to reduce its intrinsic $Q$, or by increasing the coupling to the bus waveguide to reduce its coupling $Q$. Parasitic mode coupling between non-pumped resonances can also transfer comb power from the NR to the RC, leading to additional comb power leakage through the through port (see Supplementary Information). This effect can be mitigated by engineering the NR–RC coupling to be significant only in the vicinity of the pump resonances. Most critically, the parameters of both the RC and NR must be precisely controlled to minimize direct pump transmission through the bus waveguide, thereby realizing the desired generalized critical-coupling condition^20,22^. The required agreement between design and fabrication has been achieved in our process (see Supplementary Information).

With the pump-power bottleneck relaxed, future efforts can concentrate on optimizing microcomb performance through engineering the NRs. Large-dispersion NRs within the resonant-coupler architecture will deliver large numbers of high-power comb teeth with signal-to-noise ratios suited to advanced telecom formats – potentially obviating external optical amplification^40,41^. Simultaneously, robust f-2f self-referencing will demand precise control over dispersive-wave generation^9,42–45^. Finally, just as the input port of the microcomb can be engineered to enhance pump delivery, the output spectrum itself may be sculpted via wavelength-selective couplers to meet specific application requirements.

## Materials and methods

### Impossible trinity of soliton microcombs

The conventional soliton microcombs are described by the Lugiato–Lefever equation^46^:

3 $$\frac{\partial A}{\partial T}=-\frac{{\kappa }_{{\mathrm{NR}}}}{2}A-i\delta \omega A+i\frac{{D}_{2}}{2}\frac{{\partial }^{2}A}{\partial {\phi }^{2}}+{ig}{\left|A\right|}^{2}A+\sqrt{\frac{{\kappa }_{{\mathrm{e}},{\mathrm{NR}}}{P}_{{\mathrm{in}}}}{\hslash {\omega }_{0}}}$$

where $T$ is the slow time (lab time) and $\phi$ is the angular coordinate in the moving frame. $A\left(T,\phi \right)$ corresponds to the slowly varying field amplitude, which is normalized such that ${\left|A\right|}^{2}$ corresponds to the intracavity photon number. ${D}_{2}$ is the second-order dispersion. The decay rates of NR is defined as ${\kappa }_{{\rm{NR}}}={\kappa }_{0,{\rm{NR}}}+{\kappa }_{{\rm{e}},{\rm{NR}}}$, where ${\kappa }_{0,{\rm{NR}}}$ is the intrinsic decay rates and ${\kappa }_{{\rm{e}},{\rm{NR}}}$ is the coupling rates to the waveguide. $g$ denotes the nonlinear coefficient, which is defined as $g=\frac{\hslash {\omega }_{0}^{2}c{n}_{2}}{{n}_{0}^{2}{V}_{{eff}}}$, where ${V}_{{\rm{eff}}}$ is the effective mode volume and ${n}_{2}$ is the nonlinear refractive index associated with the refractive index ${n}_{0}$. $\delta \omega$ is the pump-NR detuning and ${P}_{{\rm{in}}}$ is the input pump power. The onset of four-wave mixing occurs when ${P}_{{\rm{in}}}$ exceeds the threshold ${P}_{{\rm{th}}}$:

4 $${P}_{{\mathrm{th}}}=\frac{\hslash {\omega }_{0}{\kappa }_{{\mathrm{NR}}}^{3}}{8g{\kappa }_{\mathrm{e},{\mathrm{NR}}}}$$

However, sustaining solitons at a given detuning $\delta \omega$ requires additional pump power,

5 $${P}_{{\rm{in}}}\ge \frac{16}{{\pi }^{2}}\times \frac{\delta \omega {P}_{{\rm{th}}}}{{\kappa }_{{\rm{NR}}}}$$

The detuning is a key parameter determining the comb span:

6 $$\varDelta {f}_{3{\rm{dB}}}=\frac{1.763}{{\pi }^{2}}\times \sqrt{-\frac{2{n}_{0}\delta \omega }{c{\beta }_{2}}}$$

where ${\beta }_{2}=-\frac{{n}_{0}{D}_{2}}{c{D}_{1}^{2}}$ is the group velocity dispersion coefficient, with ${D}_{1}$ denoting the FSR in angular frequency. Combining Eqs. 5,6 and using the approximation ${D}_{1}\approx 2\pi {f}_{{\rm{r}}}$, we derive a lower bound on the pump power required to support a soliton microcomb with a specified bandwidth:

7 $${P}_{{\rm{in}}}\ge -\frac{{\pi }^{2}}{{1.763}^{2}}\times \frac{{\kappa }_{{\rm{NR}}}{\beta }_{2}}{{\eta }_{{\rm{NR}}}\gamma }\times \frac{\varDelta {f}_{3{\rm{dB}}}^{2}}{{f}_{{\rm{r}}}}$$

where $\gamma =\frac{{\omega }_{0}{n}_{2}}{c{A}_{{\rm{eff}}}}$ is the nonlinear parameter, with ${A}_{{\rm{eff}}}$ denoting the effective mode area. Additionally, the central-tooth power (${P}_{{\rm{c}}}$) of soliton microcombs can be expressed as:

8 $${P}_{{\rm{c}}}=-{\pi }^{2}\times \frac{{\kappa }_{{\rm{e}},{\rm{NR}}}{\beta }_{2}{f}_{{\rm{r}}}}{\gamma }$$

By eliminating the material-dependent terms (${\beta }_{2},\gamma$) through dividing Eq. 7 by Eq. 8, we obtain a constraint among central-tooth power, 3 dB bandwidth and repetition rate under limited pump power– referred to as the “impossible trinity”,

9 $$\frac{{P}_{{\rm{c}}}\varDelta {f}_{3{\rm{dB}}}^{2}}{{f}_{{\rm{r}}}^{2}}\le {1.763}^{2}\times {\eta }_{{\rm{NR}}}^{2}{P}_{{\rm{in}}}\approx 3.1\times {\eta }_{{\rm{NR}}}^{2}{P}_{{\rm{in}}}$$

A detailed derivation is provided in the Supplementary Information.

### Device fabrication

The Si_3_N_4_ coupled microresonators are fabricated on a 4-inch wafer through subtractive processes^47^. Initially, a 786 nm-thick Si_3_N_4_ film is deposited in two steps onto a wet-oxidized silicon substrate featuring stress-release patterns. Electron beam lithography is used to define the pattern, followed by dry etching to transfer the resist pattern to the Si_3_N_4_ film. The wafer is then annealed at 1200 °C to remove residual N-H and Si-H bonds from the Si_3_N_4_ film. SiO_2_ cladding is deposited, followed by a second annealing step for densifying the film. A lift-off process is then used to define the heater patterns. Finally, the wafer is diced into 5 mm $\times$ 5 mm chips.

### Device characterization

Our work involves five devices for microcomb generation. Device 1 is used for soliton generation in the waveguide-coupled NR (Fig. 2h). Device 2 is employed to demonstrate high-power ultra-broadband soliton microcombs (Fig. 2g). Device 3 supports an octave-spanning microcomb at ${f}_{{\rm{r}}}$ of 100 GHz (Fig. 3a). Device 4 enables an octave-spanning microcomb at ${f}_{{\rm{r}}}$ of 25 GHz (Fig. 3c). Device 5 realizes hybrid-integrated soliton microcombs (Fig. 4a). Devices 2–5 adopt the RC architecture.

For Device 1, the NR with waveguide width 1.8 μm has ${Q}_{0}=7.29\times {10}^{6}$, ${Q}_{{\rm{e}}}=3.83\times {10}^{6}$. For Device 2, the NR (1.8 μm) has ${Q}_{0}=6.48\times {10}^{6}$, ${Q}_{{\rm{e}}}=3.81\times {10}^{6}$, while the RC (1.5 μm) has ${Q}_{0}=6.75\times {10}^{6}$, ${Q}_{{\rm{e}}}=0.38\times {10}^{6}$. Revealed by avoided crossings, the inter-resonator coupling rate is $G/2\pi =1.65$ GHz. For Device 3, the NR (3.4 μm) has ${Q}_{0}=20.64\times {10}^{6}$, ${Q}_{{\rm{e}}}=3.29\times {10}^{6}$, while the RC (1.8 μm) has ${Q}_{0}=6.67\times {10}^{6}$, ${Q}_{{\rm{e}}}=0.25\times {10}^{6}$. The inter-resonator coupling rate is $G/2\pi =0.73$ GHz. For Device 4, the NR (3.8 μm) has ${Q}_{0}=9.56\times {10}^{6}$, ${Q}_{{\rm{e}}}=9.56\times {10}^{6}$, while the RC (1.3 μm) is strongly overcoupled, making its Q factor difficult to characterize. The inter-resonator coupling rate is $G/2\pi =0.32$ GHz. For Device 5, the NR (2.5 μm) has ${Q}_{0}=14.52\times {10}^{6}$, ${Q}_{{\rm{e}}}=21.44\times {10}^{6}$, while the RC (1 μm) has ${Q}_{0}=2.79\times {10}^{6}$, ${Q}_{{\rm{e}}}=0.2\times {10}^{6}$. The inter-resonator coupling rate is $G/2\pi =0.47$ GHz.

### Characterization of repetition-rate noise and laser noise

The repetition-rate phase noise of the 100 GHz soliton microcomb is characterized using a multi-frequency delayed self-heterodyne setup^48^ (see Figure S11 in Supplementary Information). Two comb lines are selected by a programmable optical filter and amplified by an EDFA. One path is frequency-shifted, while the other is temporally delayed. After recombination, the signals include both the original and frequency-shifted components of the selected comb lines. These are separated using a fiber Bragg grating and individually detected by two photodetectors. The beatnote phases ${\varPhi }_{i},{\varPhi }_{j}$ for comb modes $i$ and $j$ are simultaneously extracted via the Hilbert transform of the oscilloscope traces. The repetition-rate phase noise is derived from the power spectral density (PSD) of the phase difference between the two beatnotes,

10 $${S}_{{\varphi }_{{\rm{r}}}}\left(f\right)=\frac{{PSD}\left[{\varPhi }_{i}-{\varPhi }_{j}\right]}{{\left(i-j\right)}^{2}}\frac{1}{4{\sin }^{2}\left(\pi f{\tau }_{{\rm{d}}}\right)}$$

where ${\tau }_{{\rm{d}}}$ denotes the time delay between the two interferometer arms. The phase noise of the pump laser is measured using the same delayed self-heterodyne interferometer. Unlike the phase-noise measurement of the repetition rate, the beatnote phase noise (${S}_{{\rm{b}}}\left(f\right)$) is directly analyzed with a phase noise analyzer (PNA; Rohde & Schwarz FSWP50). The laser’s phase noise is then obtained from

11 $${S}_{{\varphi }_{{\rm{l}}}}\left(f\right)=\frac{{S}_{{\rm{b}}}\left(f\right)}{4{\sin }^{2}\left(\pi f{\tau }_{{\rm{d}}}\right)}$$

To prevent singularities at offset frequencies where $\sin \pi f{\tau }_{{\rm{d}}}=0$, a cut-off frequency is set at $1/{\tau }_{{\rm{d}}}$ and only data points at offset frequencies of $\left(N+1/2\right)/{\tau }_{{\rm{d}}}$, where $N$ is an integer, are retained for analysis.

The repetition rate of the 25 GHz soliton microcomb is directly measured using a high-speed photodetector connected to an electrical spectrum analyzer (see Figure. S11 in Supplementary Information). Before detection, residual pump light is suppressed using a notch filter, and the comb is amplified to 2 mW. The phase noise is further characterized using a phase noise analyzer.

## Supplementary information

Supplementary information: Power-eﬀicient ultra-broadband soliton microcombs in resonantly-coupled microresonators

## Data Availability

The data that support the plot within this paper and other findings of this study are available upon reasonable request.

## Code availability

The codes that support the findings of this study are available upon reasonable request.
