# Supplementary material for: Power-efficient ultra-broadband soliton microcombs in resonantly-coupled microresonators
Source: Light Sci Appl. 2026 Mar 30;15:185. doi: 10.1038/s41377-026-02186-9 (PMC13033455; doi:10.1038/s41377-026-02186-9)
Supplement: Supplementary file 1 — Supplementary information: Power-eﬀicient ultra-broadband soliton microcombs in resonantly-coupled microresonators [file 41377_2026_2186_MOESM1_ESM.pdf]

Supplementary information: Power-efficient ultra-broadband soliton microcombs in resonantly-coupled microresonators

Kaixuan Zhu[1*], Xinrui Luo[1*], Yuanlei Wang[1,2*], Ze Wang[1*], Tianyu Xu[1], Du Qian[1], Yinke Cheng[1,2], Junqi Wang[1], Haoyang Luo[1], Yanwu Liu[1], Xing Jin[1], Zhenyu Xie[1], Xin Zhou[2], Min Wang[2], Jian-Fei Liu[2], Xuening Cao[2], Ting Wang[2], Shui-Jing Tang[3], Qihuang Gong[1,4,5], Bei-Bei Li[2], and Qi-Fan Yang[1,4,5†]
[1]State Key Laboratory for Artificial Microstructure and Mesoscopic Physics and Frontiers Science Center for Nano-optoelectronics, School of Physics, Peking University, Beijing 100871, China
[2]Beijing National Laboratory for Condensed Matter Physics, Institute of Physics, Chinese Academy of Sciences, Beijing 100190, China
[3]National Biomedical Imaging Center, College of Future Technology, Peking University, Beijing, 100871, China
[4]Peking University Yangtze Delta Institute of Optoelectronics, Nantong, Jiangsu 226010, China
[5]Collaborative Innovation Center of Extreme Optics, Shanxi University, Taiyuan 030006, China
[*]These authors contributed equally to this work.
[†]Corresponding author: leonardoyoung@pku.edu.cn
(Dated: December 31, 2025)

CONTENTS

I. Theory for waveguide-coupled microresonators 2

II. Theory for resonantly-coupled microresonators 4
A. Theoretical model and master equations 4
B. Effective pump power 5
C. Soliton existence range 6
D. Effective pump power in the modulation instability regime 7
E. Phase diagram 7
F. Simulation based on complete model for coupled resonators 10

III. Device characterization 12
A. Dispersion 12
B. Coupling design and experimental verification of coupled microresonators 14

IV. Additional experimental results 15
A. Characterization of tuning process 15
B. Resonant frequencies of coupled microresonators 15
C. Optical spectrum 16
D. Autocorrelation 17
E. Measurement of the repetition rate 17
F. Coherence of the dispersive wave 18
G. Setup for coherence characterization of octave-spanning soliton microcombs. 19

References 20

I. THEORY FOR WAVEGUIDE-COUPLED MICRORESONATORS

We begin by analyzing a conventional configuration in which a nonlinear microresonator (NR) is evanescently coupled to a single bus waveguide. When the NR is pumped by with a continuous-wave (CW) laser, the dynamics are governed by the Lugiato–Lefever equation (LLE)[1]:

$$\frac{\partial A}{\partial T} = -\frac{\kappa_{\mathrm{NR}}}{2}A - i\delta\omega_{\mathrm{NR}}A + i\frac{D_{2,\mathrm{NR}}}{2}\frac{\partial^2 A}{\partial \phi^2} + ig_{\mathrm{NR}}|A|^2A + \sqrt{\frac{\kappa_{\mathrm{e,NR}}P_{\mathrm{in}}}{\hbar\omega_0}}, \tag{S1}$$

where T is the slow time (lab time) and ϕ is the angular coordinate in the moving frame. $A(T,\phi)$ corresponds to the slowly varying field amplitude, which is normalized such that $|A|^2$ corresponds to the intracavity photon number. $D_{2,\mathrm{NR}}$ is the second-order dispersion in the NR. The decay rates of NR is defined as $\kappa_{\mathrm{NR}} = \kappa_{0,\mathrm{NR}} + \kappa_{\mathrm{e,NR}}$, where $\kappa_{0,\mathrm{NR}}$ is the intrinsic decay rates and $\kappa_{\mathrm{e,NR}}$ is the coupling rates to the waveguide. g_{NR} denotes the nonlinear coefficient of the NR, which is defined as $g_{\mathrm{NR}} = \frac{\hbar\omega_0^2 c n_2}{n_0^2 V_{\mathrm{eff,NR}}}$, where $V_{\mathrm{eff,NR}}$ is the effective mode volume of NR and n_2 is the nonlinear refractive index associated with the refractive index n_0. $\delta\omega_{\mathrm{NR}}$ is the pump-NR detuning and P_{in} is the pump power.

The approximate solution to the single-soliton state can be expressed as,

$$A_{\mathrm{tot}} = A_{\mathrm{cw}} + A_{\mathrm{sol}}, \tag{S2}$$

where A_{cw} is the continuous-wave background and A_{sol} denotes the hyperbolic secant waveform in the time domain. To derive the analytical expression for A_{sol}, we first neglect the loss and pump terms, which can be regarded as small perturbations. In this case, the Lugiato–Lefever equation (LLE) reduces to the unperturbed soliton equation,

$$\frac{\partial A}{\partial T} = -i\delta\omega_{\mathrm{NR}}A + i\frac{D_{2,\mathrm{NR}}}{2}\frac{\partial^2 A}{\partial \phi^2} + ig_{\mathrm{NR}}|A|^2A, \tag{S3}$$

with a steady-state solution in the form of $A = B\mathrm{sech}(\phi/\phi_\tau)$. Here, B is the soliton amplitude, ϕ_τ is the pulse width in ϕ coordinate. Substituting this ansatz into eq. S3 yields,

$$\left(g_{\mathrm{NR}}B^2 - \frac{D_{2,\mathrm{NR}}}{\phi_\tau^2}\right)\mathrm{sech}^2\left(\frac{\phi}{\phi_\tau}\right) + \frac{D_{2,\mathrm{NR}}}{2\phi_\tau^2} - \delta\omega_{\mathrm{NR}} = 0. \tag{S4}$$

For the above equation to hold for any ϕ, the coefficients of the $\mathrm{sech}^2(\phi/\phi_\tau)$ term and the constant term must both vanish, leading to

$$g_{\mathrm{NR}}B^2 = \frac{D_{2,\mathrm{NR}}}{\phi_\tau^2}, \tag{S5}$$

$$\frac{D_{2,\mathrm{NR}}}{2\phi_\tau^2} = \delta\omega_{\mathrm{NR}}. \tag{S6}$$

Thus, the parameters B and ϕ_τ can be written as

$$B = \sqrt{\frac{2\delta\omega_{\mathrm{NR}}}{g_{\mathrm{NR}}}}, \phi_\tau = \sqrt{\frac{D_{2,\mathrm{NR}}}{2\delta\omega_{\mathrm{NR}}}} \tag{S7}$$

We next consider the influence of cavity loss and external pumping. The soliton ansatz of the LLE (eq. S1) can be expressed as

$$A_{\mathrm{sol}} = B\,\mathrm{sech}(\phi/\phi_\tau)e^{i\varphi_0} = \sqrt{\frac{2\delta\omega_{\mathrm{NR}}}{g_{\mathrm{NR}}}}\mathrm{sech}\left(\sqrt{\frac{2\delta\omega_{\mathrm{NR}}}{D_{2,\mathrm{NR}}}}\phi\right)e^{i\varphi_0}, \tag{S8}$$

where φ_0 specifies the soliton's phase relative to the pump. To determine φ_0, we employ the moment analysis method. The energy of the soliton is defined as

$$E_{\mathrm{sol}} = \frac{1}{2\pi}\int_{-\pi}^{\pi}|A_{\mathrm{sol}}(\phi,t)|^2\,\mathrm{d}\phi = \frac{B^2\phi_\tau}{\pi}, \tag{S9}$$

whose equation of motion can be written as

$$\frac{dE_{\mathrm{sol}}}{dT} = -\kappa_{\mathrm{NR}} E_{\mathrm{sol}} + \frac{1}{2\pi}\sqrt{\frac{\kappa_{\mathrm{e,NR}} P_{\mathrm{in}}}{\hbar\omega_0}} \int_{-\pi}^{\pi} (A_{\mathrm{sol}} + A^*_{\mathrm{sol}})\, \mathrm{d}\phi = -\frac{B^2 \phi_\tau \kappa_{\mathrm{NR}}}{\pi} + B\phi_\tau \sqrt{\frac{\kappa_{\mathrm{e,NR}} P_{\mathrm{in}}}{\hbar\omega_0}} \cos(\varphi_0). \tag{S10}$$

At steady state, the soliton energy should be invariant ($\frac{dE_{\mathrm{sol}}}{dT} = 0$). Substituting Eq. S7 gives,

$$\cos(\varphi_0) = \frac{B\kappa_{\mathrm{NR}}}{\pi}\sqrt{\frac{\hbar\omega_0}{\kappa_{\mathrm{e,NR}} P_{\mathrm{in}}}} = \frac{4}{\pi}\sqrt{\frac{\delta\omega_{\mathrm{NR}} P_{\mathrm{th,NR}}}{\kappa_{\mathrm{NR}} P_{\mathrm{in}}}}, \tag{S11}$$

where $P_{\mathrm{th,NR}}$ is the parametric oscillation threshold, defined as $P_{\mathrm{th,NR}} = \frac{\hbar\omega_0 \kappa^3_{\mathrm{NR}}}{8 g_{\mathrm{NR}} \kappa_{\mathrm{e,NR}}}$. The constraint $\cos(\varphi_0) \leq 1$ implies the minimum pump power required to support a soliton at a given detuning:

$$P_{\mathrm{in}} \geq \frac{16}{\pi^2} \times \frac{\delta\omega_{\mathrm{NR}} P_{\mathrm{th,NR}}}{\kappa_{\mathrm{NR}}}. \tag{S12}$$

The spectral envelope of the soliton microcomb is given by the Fourier transform:

$$\tilde{A}_{\mathrm{sol}}(\mu) = \mathscr{F}[A_{\mathrm{sol}}(\phi)] = \sqrt{\frac{D_{2,\mathrm{NR}}}{4 g_{\mathrm{NR}}}} \mathrm{sech}\left(\frac{\pi\mu}{2}\sqrt{\frac{D_{2,\mathrm{NR}}}{2\delta\omega_{\mathrm{NR}}}}\right) e^{i\varphi_0}, \tag{S13}$$

where μ is the mode number relative to the pump. Using the relation for comb frequencies,

$$\omega_\mu = \omega_{\mathrm{p}} + \mu\omega_{\mathrm{r}}, \tag{S14}$$

where ω_{p} is the pump frequency and ω_{r} is the repetition frequency. Since ω_{r} is close to the free spectral range (FSR, $D_{1,\mathrm{NR}}$), $\tilde{A}_{\mathrm{sol}}(\mu)$ becomes:

$$\tilde{A}_{\mathrm{sol}}(\omega_\mu - \omega_{\mathrm{p}}) = \sqrt{\frac{D_{2,\mathrm{NR}}}{4 g_{\mathrm{NR}}}} \mathrm{sech}\left(\frac{\omega_\mu - \omega_{\mathrm{p}}}{\Delta\omega}\right) e^{i\varphi_0} \quad \text{with} \quad \Delta\omega = \frac{2 D_{1,\mathrm{NR}}}{\pi}\sqrt{\frac{2\delta\omega_{\mathrm{NR}}}{D_{2,\mathrm{NR}}}}. \tag{S15}$$

This equation can be reformulated in terms of the group velocity dispersion coefficient $\beta_{2,\mathrm{NR}} = -\frac{n_0 D_{2,\mathrm{NR}}}{c D^2_{1,\mathrm{NR}}}$ and the nonlinear parameter $\gamma_{\mathrm{NR}} = \frac{\omega_0 n_2}{c A_{\mathrm{eff,NR}}}$, with $A_{\mathrm{eff,NR}}$ denoting the effective mode area of NR. Using the approximation $D_{1,\mathrm{NR}} \approx 2\pi f_{\mathrm{r}}$, where f_{r} is the soliton repetition rate, the soliton spectrum takes the form:

$$\tilde{A}_{\mathrm{sol}}(\omega_\mu - \omega_{\mathrm{p}}) = \pi\sqrt{-\frac{\beta_{2,\mathrm{NR}} f_{\mathrm{r}}}{\hbar\omega_0 \gamma_{\mathrm{NR}}}} \mathrm{sech}\left(\frac{\omega_\mu - \omega_{\mathrm{p}}}{\Delta\omega}\right) e^{i\varphi_0} \quad \text{with} \quad \Delta\omega = \frac{2}{\pi}\sqrt{-\frac{2 n_0 \delta\omega_{\mathrm{NR}}}{c\beta_{2,\mathrm{NR}}}}. \tag{S16}$$

Equation S16 reveals two key spectral metrics of soliton microcombs:

1. Central-tooth power (P_{c}):

$$P_{\mathrm{c}} = \hbar\omega_0 \kappa_{\mathrm{e,NR}} |\tilde{A}_{\mathrm{sol}}(0)|^2 = -\pi^2 \times \frac{\kappa_{\mathrm{e,NR}} \beta_{2,\mathrm{NR}} f_{\mathrm{r}}}{\gamma_{\mathrm{NR}}}. \tag{S17}$$

2. 3-dB bandwidth ($\Delta f_{3\mathrm{dB}}$):

$$\Delta f_{3\mathrm{dB}} = 1.763 \times \frac{\Delta\omega}{2\pi} = \frac{1.763}{\pi^2} \times \sqrt{-\frac{2 n_0 \delta\omega_{\mathrm{NR}}}{c\beta_{2,\mathrm{NR}}}}. \tag{S18}$$

Here, the factor 1.763 corresponds to $2\cosh^{-1}(\sqrt{2})$, which converts the spectral width from 1/e to the full width at half maximum for a sech^2-shaped spectrum. Combining Eqs. S12, S18, we obtain a lower bound on the pump power necessary to sustain a soliton microcomb with a specified bandwidth:

$$P_{\mathrm{in}} \geq -\frac{\pi^2}{1.763^2} \times \frac{\kappa_{\mathrm{NR}} \beta_{2,\mathrm{NR}}}{\eta_{\mathrm{NR}} \gamma_{\mathrm{NR}}} \times \frac{\Delta f^2_{3\mathrm{dB}}}{f_{\mathrm{r}}}, \tag{S19}$$

where $\eta_{\mathrm{NR}} = \kappa_{\mathrm{e,NR}}/\kappa_{\mathrm{NR}}$ is the loading factor. Equation S19 implies that the pump power requirement increases quadratically with spectral bandwidth and decreases with repetition rate. Dividing Eq. S19 by Eq. S17 removes material-specific parameters ($\beta_{2,\mathrm{NR}}, \gamma_{\mathrm{NR}}$) and yields an intrinsic constraint—termed the "impossible trinity"—that links the 3-dB bandwidth, repetition rate, and central-tooth power under available pump power,

$$\frac{P_{\mathrm{c}}\Delta f_{\mathrm{3dB}}^2}{f_{\mathrm{r}}^2} \leq 1.763^2 \times \eta_{\mathrm{NR}}^2 P_{\mathrm{in}} \approx 3.1 \times \eta_{\mathrm{NR}}^2 P_{\mathrm{in}}. \tag{S20}$$

II. THEORY FOR RESONANTLY-COUPLED MICRORESONATORS

A. Theoretical model and master equations

The system comprising two coupled microresonators is described by a set of coupled LLEs:

$$\frac{\partial B}{\partial T} = -\frac{\kappa_{\mathrm{RC}}}{2}B - i\delta\omega_{\mathrm{RC}}B - \mathcal{F}\left[iD_{\mathrm{int,RC}}(\mu)\tilde{B}_\mu\right] + ig_{\mathrm{RC}}|A|^2A + iGA + \sqrt{\frac{\kappa_{\mathrm{e,RC}}P_{\mathrm{in}}}{\hbar\omega_0}}, \tag{S21}$$

$$\frac{\partial A}{\partial T} = -\frac{\kappa_{\mathrm{NR}}}{2}A - i\delta\omega_{\mathrm{NR}}A - \mathcal{F}\left[iD_{\mathrm{int,NR}}(\mu)\tilde{A}_\mu\right] + ig_{\mathrm{NR}}|A|^2A + iGB, \tag{S22}$$

where $B(T,\phi)$and $A(T,\phi)$ denote the slowly varying field amplitude of the resonant coupler (RC) and the nonlinear resonator (NR), respectively. The two quantities are normalized such that $|B|^2$ and $|A|^2$ correspond to the intracavity photon number. $\tilde{B}_\mu(T)$ and $\tilde{A}_\mu(T)$ are the optical field of the μ-th mode, obtained from $B(T,\phi)$ and $A(T,\phi)$ via the Fourier transform, respectively. Definitions of these quantities: $\kappa_{\mathrm{RC(NR)}}, g_{\mathrm{RC(NR)}}$ are consistent with that in Section I. $D_{\mathrm{int,RC}}$ and $D_{\mathrm{int,NR}}$ are the integrated dispersion of the RC and NR, which are defined relative to the NR as $D_{\mathrm{int,RC(NR)}} = \omega_{\mu,\mathrm{NR(RC)}} - \omega_{0,\mathrm{NR(RC)}} - \mu D_{1,\mathrm{NR}}$. The coupling strength between the microresonators is given by a real number G. $\delta\omega_{\mathrm{RC(NR)}}$ is the pump-RC (NR) detuning. P_{in} is the pump power on the RC.

Notably, the NR and the RC in our configuration are evanescently coupled, with the coupling strength far below half of their average FSR[2]. In this regime, it is sufficient to consider coupling only between the 0-th modes of the two resonators, as other mode interactions have negligible impacts on the comb state in the NR. Furthermore, the Kerr nonlinearity in the RC can also be neglected as its intracavity energy remains well below the threshold for parametric oscillation. Thus, the RC is reduced to a single-mode linear microresonator. The resulting simplified model is given below when neglecting the Raman effect as well as third and higher-order dispersion terms of the NR:

$$\frac{db_0}{dT} = -\frac{\kappa_{\mathrm{RC}}}{2}b_0 - i\delta\omega_{\mathrm{RC}}b_0 + iGa_0 + \sqrt{\frac{\kappa_{\mathrm{e,RC}}P_{\mathrm{in}}}{\hbar\omega_0}}, \tag{S23}$$

$$\frac{\partial A}{\partial T} = -\frac{\kappa_{\mathrm{NR}}}{2}A - i\delta\omega_{\mathrm{NR}}A + i\frac{D_{2,\mathrm{NR}}}{2}\frac{\partial^2 A}{\partial\phi^2} + ig_{\mathrm{NR}}|A|^2A + iGb_0, \tag{S24}$$

where b_0 and a_0 denote the field amplitude in 0-th mode of the RC and NR, respectively.

For convenience, we normalize the coupled LLE as follows:

$$\frac{d\psi_{\mathrm{RC}}}{d\tau} = -(\kappa_{\mathrm{r}} + i\zeta_{\mathrm{RC}})\psi_{\mathrm{RC}} + ig_{\mathrm{c}}\psi_{0,\mathrm{NR}} + f_{\mathrm{RC}}, \tag{S25}$$

$$\frac{\partial\psi_{\mathrm{NR}}}{\partial\tau} = -(1 + i\zeta_{\mathrm{NR}})\psi_{\mathrm{NR}} + id_{2,\mathrm{NR}}\frac{\partial^2\psi_{\mathrm{NR}}}{\partial\phi^2} + i|\psi_{\mathrm{NR}}|^2\psi_{\mathrm{NR}} + ig_{\mathrm{c}}\psi_{\mathrm{RC}}, \tag{S26}$$

where $\tau = \frac{\kappa_{\mathrm{NR}}}{2}T$, $A = \sqrt{\frac{\kappa_{\mathrm{NR}}}{2g_{\mathrm{NR}}}}\psi_{\mathrm{NR}}, b_0 = \sqrt{\frac{\kappa_{\mathrm{NR}}}{2g_{\mathrm{NR}}}}\psi_{\mathrm{RC}}, a_0 = \sqrt{\frac{\kappa_{\mathrm{NR}}}{2g_{\mathrm{NR}}}}\psi_{0,\mathrm{NR}}, \zeta_{\mathrm{NR}} = \frac{2\delta\omega_{\mathrm{NR}}}{\kappa_{\mathrm{NR}}}, \zeta_{\mathrm{RC}} = \frac{2\delta\omega_{\mathrm{RC}}}{\kappa_{\mathrm{NR}}}, \kappa_{\mathrm{r}} = \frac{\kappa_{\mathrm{RC}}}{\kappa_{\mathrm{NR}}}, g_{\mathrm{c}} = \frac{2G}{\kappa_{\mathrm{NR}}}, d_{2,\mathrm{NR}} = \frac{D_{2,\mathrm{NR}}}{\kappa_{\mathrm{NR}}}, f_{\mathrm{RC}} = \sqrt{\frac{8g_{\mathrm{NR}}\kappa_{\mathrm{e,RC}}P_{\mathrm{in}}}{\kappa_{\mathrm{NR}}^3\hbar\omega_0}}$. The normalized pump power on RC can be described as

$$f_{\mathrm{RC}}^2 = \frac{P_{\mathrm{in}}}{P_{\mathrm{th,NR}}}\cdot\frac{\kappa_{\mathrm{e,RC}}}{\kappa_{\mathrm{e,NR}}} = f_{\mathrm{NR}}^2\cdot\frac{\kappa_{\mathrm{e,RC}}}{\kappa_{\mathrm{e,NR}}}. \tag{S27}$$

Here, $P_{\mathrm{th,NR}}$ is the parametric oscillation threshold of the NR when coupled from the bus waveguide, as defined in Section I, and f_{NR}^2 denotes the corresponding normalized pump power for the NR.

B. Effective pump power

As shown in Eq. S26, the power injected into NR is predominantly governed by the coupling term $ig_{\mathrm{c}}\psi_{\mathrm{RC}}$. Therefore, we define the effective pump term as:

$$f_{\mathrm{eff}} = ig_{\mathrm{c}}\psi_{\mathrm{RC}}, \tag{S28}$$

such that Eq. S26 recovers the the standard form of the LLE. $|f_{\mathrm{eff}}^2|$ can be estimated by analyzing the steady-state continuous-wave solution of the coupled LLE. To this end, we first consider the optical field of the 0-th mode of the NR, given by,

$$\psi_{0,\mathrm{NR}} \approx \frac{ig_{\mathrm{c}}\psi_{\mathrm{RC}}}{1 + i\zeta_{\mathrm{NR}}}. \tag{S29}$$

Inserting this into Eq. S25 and considering $\frac{\partial\psi_{\mathrm{RC}}}{\partial\tau} = 0$, we can get:

$$f_{\mathrm{RC}} = (\kappa_{\mathrm{r}} + i\zeta_{\mathrm{RC}})\psi_{\mathrm{RC}} + \frac{g_{\mathrm{c}}^2}{1 + i\zeta_{\mathrm{NR}}}\psi_{\mathrm{RC}}. \tag{S30}$$

Taking the modulus, we obtain the intracavity power of RC:

$$|\psi_{\mathrm{RC}}|^2 = \frac{f_{\mathrm{RC}}^2}{(\kappa_{\mathrm{r}} + \frac{g_{\mathrm{c}}^2}{1+\zeta_{\mathrm{NR}}^2})^2 + (\zeta_{\mathrm{RC}} - \frac{g_{\mathrm{c}}^2\zeta_{\mathrm{NR}}}{1+\zeta_{\mathrm{NR}}^2})^2}. \tag{S31}$$

Thus, the effective pump power for the NR is given by

$$|f_{\mathrm{eff}}^2| = g_{\mathrm{c}}^2|\psi_{\mathrm{RC}}|^2 = \frac{g_{\mathrm{c}}^2 f_{\mathrm{RC}}^2}{(\kappa_{\mathrm{r}} + \frac{g_{\mathrm{c}}^2}{1+\zeta_{\mathrm{NR}}^2})^2 + (\zeta_{\mathrm{RC}} - \frac{g_{\mathrm{c}}^2\zeta_{\mathrm{NR}}}{1+\zeta_{\mathrm{NR}}^2})^2}. \tag{S32}$$

As the system transitions to the soliton state, large detuning in the NR causes, $\frac{g_{\mathrm{c}}^2}{1+\zeta_{\mathrm{NR}}^2} \to 0$, and the effective pump power converges to,

$$|f_{\mathrm{eff}}^2| = \frac{g_{\mathrm{c}}^2 f_{\mathrm{RC}}^2}{\kappa_{\mathrm{r}}^2 + \zeta_{\mathrm{RC}}^2}. \tag{S33}$$

The effective pump power is maximized when the pump is resonant with the RC ($\zeta_{\mathrm{RC}} = 0$). In this regime, the optimized effective pump power is given by:

$$|f_{\mathrm{eff}}^2| = \frac{g_{\mathrm{c}}^2 f_{\mathrm{RC}}^2}{\kappa_{\mathrm{r}}^2}. \tag{S34}$$

To quantify the advantage of employing the RC, we define an enhancement factor that compares the effective pump power delivered to the NR via the RC with the case using a conventional waveguide coupler,

$$\Gamma = \frac{|f_{\mathrm{eff}}^2|}{f_{\mathrm{NR}}^2}. \tag{S35}$$

According to Eq. S34 and Eq. S27, the enhancement factor can be calculated by

$$\Gamma = \frac{4G^2}{\kappa_{\mathrm{NR}}\kappa_{\mathrm{RC}}} \cdot \frac{\eta_{\mathrm{RC}}}{\eta_{\mathrm{NR}}}. \tag{S36}$$

The loading factors $\eta_{\mathrm{NR(RC)}} = \kappa_{\mathrm{e,NR(RC)}}/\kappa_{\mathrm{NR(RC)}}$. For efficient coupling, they are usually in the range between 0.5

to 1. Therefore, the ratio $\frac{\eta_{\mathrm{RC}}}{\eta_{\mathrm{NR}}}$ is on the order of unity, and the enhancement factor is on the order of

$$\Gamma \approx \frac{4G^2}{\kappa_{\mathrm{NR}}\kappa_{\mathrm{RC}}}. \tag{S37}$$

C. Soliton existence range

As discussed in previous works[3,4], the relationship between soliton detuning and pumping in a regular LLE is given by $\zeta \leq \frac{\pi^2 f^2}{8}$. Thus, for devices with RC, the maximum detuning range of the soliton is given by:

$$\zeta_{\mathrm{NR}} \leq \frac{\pi^2 |f_{\mathrm{eff}}^2|}{8} = \Gamma \frac{\pi^2 f_{\mathrm{NR}}^2}{8}. \tag{S38}$$

Therefore, compared with waveguide-coupled NRs, in resonantly-coupled NRs the accessible detuning for soliton states is increased by a factor of Γ. However, as the detuning of the NR becomes large, a notable decrease of the effective pump power occurs. This requires additional correction that applies to the intracavity field of the NR (Eq. S29), with contribution from the 0-th mode spectral component of the soliton. Adding it to the continuous-wave background gives:

$$\psi_{0,\mathrm{NR}} = \sqrt{\frac{d_{2,\mathrm{NR}}}{2}} e^{i\varphi} + \frac{i g_{\mathrm{c}} \psi_{\mathrm{RC}}}{1 + i\zeta_{\mathrm{NR}}} \approx \sqrt{\frac{d_{2,\mathrm{NR}}}{2}} e^{i\varphi} + \frac{g_{\mathrm{c}} \psi_{\mathrm{RC}}}{\zeta_{\mathrm{NR}}}. \tag{S39}$$

where $\sqrt{\frac{d_{2,\mathrm{NR}}}{2}} e^{i\varphi}$ corresponds to the 0-th mode spectral component of the soliton and φ corresponds to the phase of the soliton. At small detuning, the first term is negligible compared with the second term. However, at large detuning, its contribution should be considered. Substituting Eq. S39 into Eq. S25 and considering $\frac{\partial \psi_{\mathrm{RC}}}{\partial \tau} = 0$ leads to:

$$f_{\mathrm{RC}} + i g_{\mathrm{c}} \sqrt{\frac{d_{2,\mathrm{NR}}}{2}} e^{i\varphi} = (\kappa_{\mathrm{r}} + i\zeta_{\mathrm{RC}})\psi_{\mathrm{RC}} + \frac{g_{\mathrm{c}}^2}{1 + i\zeta_{\mathrm{NR}}} \psi_{\mathrm{RC}}. \tag{S40}$$

Taking the modulus of both sides gives:

$$|\psi_{\mathrm{RC}}|^2 = \frac{(f_{\mathrm{RC}} - g_{\mathrm{c}} \sqrt{\frac{d_{2,\mathrm{NR}}}{2}} \sin\varphi)^2 + g_{\mathrm{c}}^2 \frac{d_{2,\mathrm{NR}}}{2} \cos^2\varphi}{(\kappa_{\mathrm{r}} + \frac{g_{\mathrm{c}}^2}{1+\zeta_{\mathrm{NR}}^2})^2 + (\zeta_{\mathrm{RC}} - \frac{g_{\mathrm{c}}^2 \zeta_{\mathrm{NR}}}{1+\zeta_{NR}^2})^2}. \tag{S41}$$

As the detuning of the NR (ζ_{NR}) increases toward the upper boundary of the soliton existence range, we find that $\sin\varphi$ approaches 1, while $\cos\varphi$ tends to 0[4]:

$$\sin\varphi = \sqrt{\frac{8\zeta_{\mathrm{NR}}}{\pi^2 g_{\mathrm{c}}^2 |\psi_{\mathrm{RC}}|^2}} \to 1, \cos\varphi \to 0. \tag{S42}$$

Therefore, the effective pump power for large detuning is modified to:

$$|f_{\mathrm{eff}}^2| = g_{\mathrm{c}}^2 |\psi_{\mathrm{RC}}|^2 \approx g_{\mathrm{c}}^2 \frac{(f_{\mathrm{RC}} - g_{\mathrm{c}} \sqrt{\frac{d_{2,\mathrm{NR}}}{2}})^2}{\kappa_{\mathrm{r}}^2}, \tag{S43}$$

which further gives the modified the soliton existence range:

$$\zeta_{\mathrm{NR}} \leq \frac{\pi^2}{8} \frac{g_{\mathrm{c}}^2 (f_{\mathrm{RC}} - g_{\mathrm{c}} \sqrt{\frac{d_{2,\mathrm{NR}}}{2}})^2}{\kappa_{\mathrm{r}}^2} = \Gamma \frac{\pi^2 f_{\mathrm{NR}}^2}{8} (1 - \frac{g_{\mathrm{c}} \sqrt{\frac{d_{2,\mathrm{NR}}}{2}}}{f_{\mathrm{RC}}})^2. \tag{S44}$$

When the coupling strength g_{c} is moderate, the ratio $\frac{g_{\mathrm{c}}\sqrt{d_{2,\mathrm{NR}}/2}}{f_{\mathrm{RC}}}$ remains on the order of 0.1, and the enhancement factor remains nearly unaffected. As the coupling strength g_{c} increases such that $g_{\mathrm{c}}\sqrt{d_{2,\mathrm{NR}}/2}$ approaches f_{RC},

the enhancement factor for the maximum detuning enabled by the RC configuration begins to diminish due to perturbations from the 0-th mode of soliton. This effect can be interpreted as an effective nonlinear loss induced by the soliton. It will be seen that such a refined theory exhibits better agreement with numerical simulations, as illustrated in Section II E.

D. Effective pump power in the modulation instability regime

The derivation above, which employs the coupled LLEs under steady CW conditions, captures the key physics of our system. However, this description assumes that the NR remains in either the CW or soliton state, where the intracavity field is well approximated by the steady-state solution. In MI regime, this assumption breaks down, as no well-defined steady-state solution exists for the intracavity field, making it difficult to directly determine f_{eff} using the previous approach. Nevertheless, once exceeding the parametric oscillation threshold, the zero-mode energy in the NR ($|\psi_{0,\mathrm{NR}}|^2$) fluctuates around 1, which is validated by the following simulations. This allows us to approximate f_{eff} as follows,

$$f_{\mathrm{eff}}^2 \approx 1 + (\zeta_{\mathrm{NR}} - 1)^2 \tag{S45}$$

Coincidentally, this result also corresponds to the boundary that separates the MI and soliton regions in the single-cavity phase diagram[5,6].

E. Phase diagram

Here, we present the protocols to generate ultra-broadband soliton microcombs. The NR supports multiple optical states, yet direct access to the soliton state from the CW regime is unattainable[4] (Fig. S1a). Instead, soliton formation is initiated via modulation instability (MI), which in waveguide-coupled NRs is reached by precisely tuning the pump from the blue-detuned to the red-detuned side of the resonance (Fig. S1b). This MI-to-soliton transition typically involves a sharp intracavity power drop and a concomitant thermo-optic blue-shift that can destabilize the soliton state; various techniques, including rapid power modulation, have been developed to address this issue[7,8].

In resonantly-coupled NRs, the tuning mechanism is fundamentally altered. Soliton initiation begins by setting the RC frequency to be blue-detuned relative to the NR, while the pump, initially red-detuned from the NR, is tuned closer until MI is triggered (Fig. S1c). The tuning process is conveniently visualized using a phase diagram defined by the relative detuning between the pump and the NR and the effective pump power delivered to the NR. In this diagram, the effective pump curve, determined by the fixed frequencies of the RC and NR during the initial tuning stage, initially leads to an increase in intracavity power as the pump is tuned closer to the NR resonance. The increased power drives the system into the monostable MI region, the only accessible pathway for transitioning from the CW state to MI[9]. As tuning continues, the effective pump power decreases, enforcing the transition into the soliton state. This tuning trajectory is effectively "backward" relative to the conventional approach. Notably, after soliton initiation using backward tuning, the pump resides on the thermally stable blue side of the hybrid resonance, which is favorable for subsequent tuning processes[10]. To extend the comb span, the detuning is subsequently increased. This requires higher effective pump power while avoiding reentry into the MI regime. We achieve this by incrementally raising the NR's resonant frequency while lowering that of the RC until their frequencies effectively swap (Fig. S1d). This maneuver shifts the effective pump curve, repositioning the soliton state to regimes of higher pump power and larger detuning. Following the swapping stage, we increase the NR's resonant frequency, while the pump laser remains aligned close to the RC resonance (Fig. S1e). The increased detuning between the pump and the NR significantly broadens the soliton microcomb spectrum to an extent typically unattainable in waveguide-coupled devices.

To validate our theoretical analysis, numerical simulations of the coupled LLE are performed using the Split-Step Fourier Transform method. Each optical mode is initially seeded with half the energy of a single photon. The normalized parameters used in Fig. S1 are: $\kappa_{\mathrm{r}} = 6.52$, $g_{\mathrm{c}} = 18.35$, $d_{2,\mathrm{NR}} = 0.002$, and $f_{\mathrm{RC}} = 9.277$.

We present the simulated evolution of states within the phase diagram, which delineates the emergence of nonlinear behavior in the NR. In Fig. S1c, the black trajectory is the theoretical trace derived from the above analytical model, while the gray trajectory depicts the simulated RC intracavity energy scaled by g_{c}^2, corresponding to the effective pump power f_{eff}^2. The two curves agree well before the system approaches the boundary of the monostable MI regime, where the intracavity energy of the NR's 0-th mode is far beyond the threshold for parametric oscillation. Continued tuning drives the system into the MI state, where the NR's 0-th mode energy abruptly drops and begins to fluctuate near 1. This sudden reduction causes the effective pump power to deviate from its original trajectory, exhibiting a sharp decline followed by oscillations near the MI–soliton boundary, as shown by the gray curve in Fig. S1c and

discussed in Section II D. The MI state persists until the effective pump power falls below a critical threshold, at which point a single soliton is generated, as illustrated in Fig. S1c.

Once the soliton is generated, the swap operation begins by gradually tuning the RC resonance toward the pump laser, as previously discussed. After the swap, the effective pump power may not have reached its maximum (red point at the gray curve in Fig. S1d). However, in the subsequent soliton broadening stage, tuning the NR mode further into the far red-detuned regime can also help approach the maximum effective pump power according to Eq. S32. This simplifies the tuning process in the soliton broadening stage and is visualized in Fig. S1e. Marked by the red star, the maximum detuning predicted by the refined theory (Section II C) agrees well with the simulated result (red dot).

It should be emphasized that the discussion above is based on a simplified model considering only the group-velocity dispersion of the NR. When additional effects such as Raman scattering and higher-order dispersion are taken into account, the maximum detuning falls well below the ideal case (Eq. S38), and so does the maximum spectral bandwidth[11,12]. In other words, the enhancement factor inferred from the experimentally observed spectral bandwidth remains substantially below the ideal value predicted by Eq. S37. A complete model that incorporates additional effects is presented in Section II F.

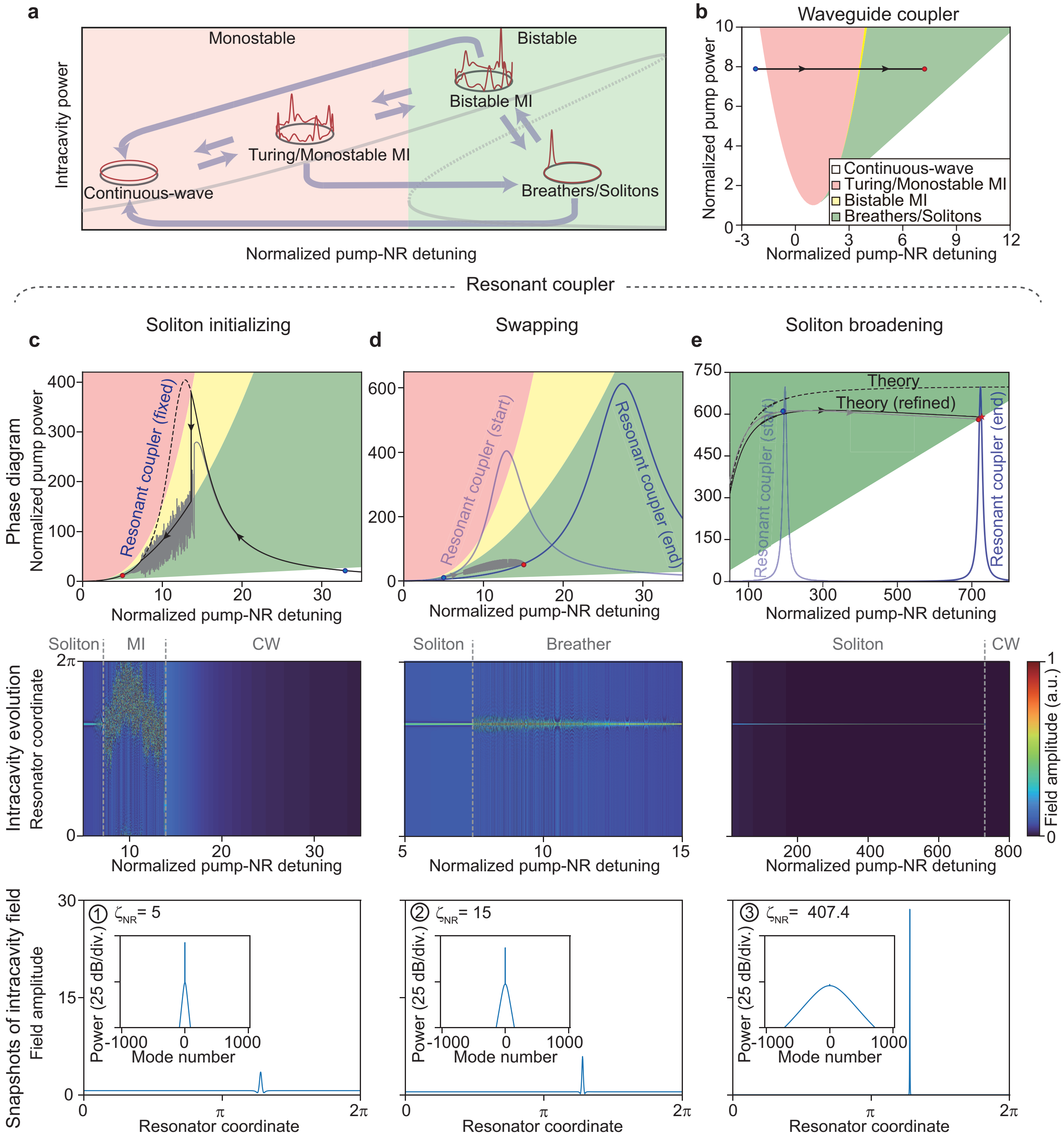

Fig. S1. **Theoretical and simulated pathways to soliton formation in phase diagrams. a,** Schematic of the permissible transitions among distinct optical states in a nonlinear resonator (NR). Background shadings indicate the monostable and bistable regimes of the equilibrium state of the NR, suggesting that modulation instability (MI) can be classified accordingly. The presence of bistability influences how the system accesses the MI state[9] and soliton state. **b,** Phase diagram for a waveguide-coupled NR, where the black trajectory delineates the evolution to soliton states at constant pump power. Blue and red dots mark the initiation and termination of the tuning process, respectively. **c–d,** Sequential stages for generating ultra-broadband solitons in a resonantly-coupled NR. Top panels: corresponding trajectories in the NR phase diagram. The blue curve represents the effective pump power delivered through the RC. The black and grey trajectories indicate the theoretical prediction and simulated evolution of the effective pump power during tuning, respectively. The simulated tuning trace begins and ends at the blue and red dots. The black dashed and solid curves in **c** represent the effective pump power during tuning, predicted by the theory and the refined theory, respectively. The maximum detuning predicted by the refined theory is marked by the red star. Middle panels: temporal evolution of the intracavity field. Bottom panels: snapshots of the intracavity field at different pump-NR detunings. Insets: optical spectra.

F. Simulation based on complete model for coupled resonators

The theoretical analysis and simulations above assume that the RC behaves as a single-mode linear microresonator. In practice, the RC can exhibit appreciable nonlinearity and dispersion and may also engage in additional mode coupling with the NR. To address these effects, we now introduce a comprehensive model that incorporates the RC's intrinsic dispersion and Kerr nonlinearity, as well as Raman scattering and higher-order dispersion in the NR:

$$\frac{\partial A}{\partial T} = -i\delta\omega_{\mathrm{NR}}A - \mathscr{F}\left[\left(\frac{\kappa_{\mathrm{NR}}(\mu)}{2} + iD_{\mathrm{int,NR}}(\mu)\right)\tilde{A}_\mu - iG(\mu)\tilde{B}_\mu\right] + ig_{\mathrm{NR}}|A|^2A + ig_{\mathrm{NR}}\,\tau_{\mathrm{R}}D_{1,\mathrm{NR}}A\frac{\partial|A|^2}{\partial\phi}, \tag{S46}$$

$$\frac{\partial B}{\partial T} = -i\delta\omega_{\mathrm{RC}}B - \mathscr{F}\left[\left(\frac{\kappa_{\mathrm{RC}}(\mu)}{2} + iD_{\mathrm{int,RC}}(\mu)\right)\tilde{B}_\mu - iG(\mu)\tilde{A}_\mu\right] + ig_{\mathrm{RC}}|B|^2B + \sqrt{\frac{\kappa_{\mathrm{e,RC}}P_{\mathrm{in}}}{\hbar\omega_0}}. \tag{S47}$$

For cases where the free spectral ranges (FSRs) of the NR and RC are close—for example, 100 GHz and 90 GHz in Devices 2 and 3—the Vernier effect between their resonances is taken into account by defining the integrated dispersion, $D_{\mathrm{int,RC(NR)}}$ in the NR-referenced frequency frame. Since the inter-resonator coupling rate G is far smaller than the FSR, we assume that only the spectrally nearest mode pairs of the NR and RC interact effectively. To simulate the optical spectra of the high-power ultra-broadband soliton microcomb presented in the main text (Device 2), we use the following parameters based on experimental measurements: $Q_{0,\mathrm{NR}} = 6.48\times10^6$, $Q_{0,\mathrm{RC}} = 6.75\times10^6$, $g_{\mathrm{NR}} = 1.37\,\mathrm{Hz}$, $g_{\mathrm{RC}} = 1.25\,\mathrm{Hz}$, $\tau_{\mathrm{R}} = 0.46\,\mathrm{fs}$ and $P_{\mathrm{in}} = 290\,\mathrm{mW}$. For the pump mode, $Q_{\mathrm{e,NR}} = 3.81\times10^6$, $Q_{\mathrm{e,RC}} = 0.38\times10^6$, $G(0)/2\pi = 1.65\,\mathrm{GHz}$. The coupling strengths– both between the waveguide and the resonators, and between the resonators themselves– are determined as a function of wavelength using finite-element simulations.

The simulated maximum pump–NR detuning is approximately 10.2 GHz. Increasing the detuning beyond this limit induces modulation instability in the RC (Fig. S2), which destabilizes the soliton microcombs in the NR.

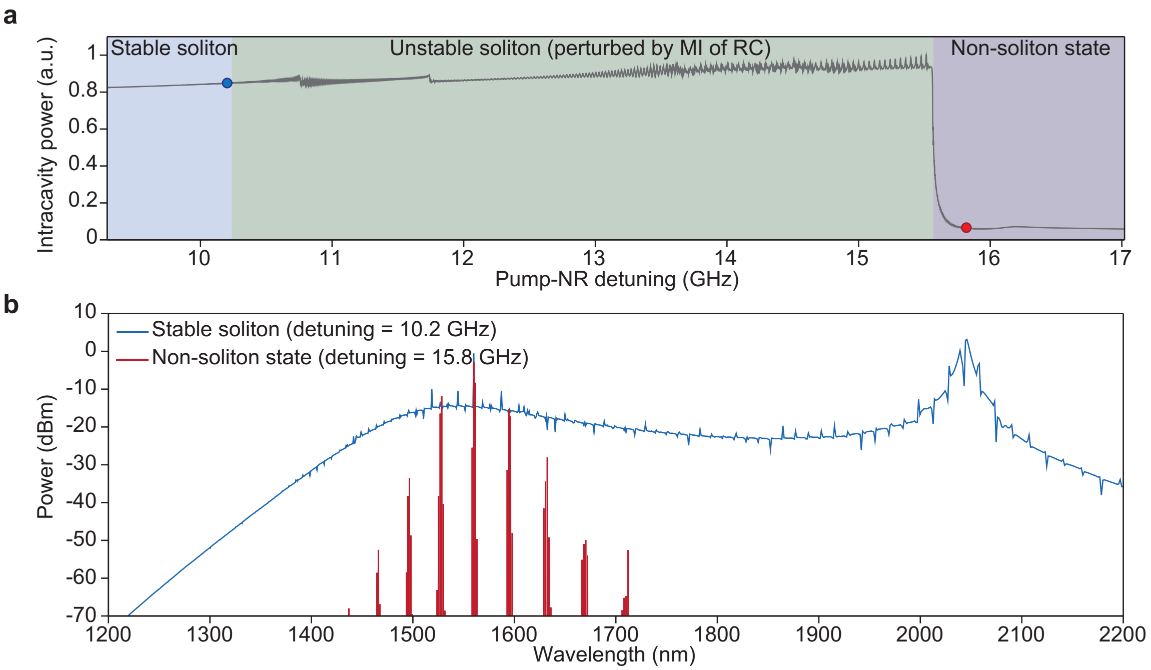

Fig. S2. **Simulation of high-power ultra-broadband soliton microcombs based on the complete model. a,** Evolution of NR's intracavity power as the pump-NR detuning is increased. **b,** Simulated optical spectra of the soliton state and non-soliton state indicated in **a**.

For cases where the FSRs of the NR and RC differ significantly—for example, 25 GHz and 115 GHz in Device 4—the large mismatch effectively suppresses Vernier effects. Accordingly, the integrated dispersion $D_{\mathrm{int,RC(NR)}}$ for nearly degenerate supermodes is unnecessary. In this regime, the inter-resonator coupling occurs near the pumped resonance, while mode crossings at other frequencies are neglected, as they do not critically influence the overall comb dynamics, which is confirmed by Helgason et al.[13]. Since the finger-shaped NR involves the Euler bends, non-

adiabatic transitions between multiple transverse modes at the interfaces between straight and bent waveguide sections introduce avoided mode crossings, particularly at shorter wavelengths. These avoid mode crossings are incorporated into $D_{\mathrm{int,NR}}$. To simulate the 25 GHz soliton microcomb, we use the following parameters based on experimental measurements: $Q_{0,\mathrm{NR}} = 9.56\times10^6$, $Q_{0,\mathrm{RC}} = 3.75\times10^6$, $g_{\mathrm{NR}} = 0.35\,\mathrm{Hz}$, $g_{\mathrm{RC}} = 1.63\,\mathrm{Hz}$, $\tau_{\mathrm{R}} = 0.46\,\mathrm{fs}$ and $P_{\mathrm{in}} = 139\,\mathrm{mW}$. For the pump mode, $Q_{\mathrm{e,NR}} = 9.56\times10^6$, $Q_{\mathrm{e,RC}} = 0.15\times10^6$, $G(0)/2\pi = 0.32\,\mathrm{GHz}$.

The simulated spectrum is presented in Fig. S3. The red arrow indicates the dispersive waves originating from higher-order dispersion, while the secondary spectral peaks are attributed to avoided mode crossings. During the simulation, no parametric oscillations are observed in the RC. The overall comb span is constrained by the spectral-envelope shift caused by Raman self-frequency shift and dispersive-wave recoil.

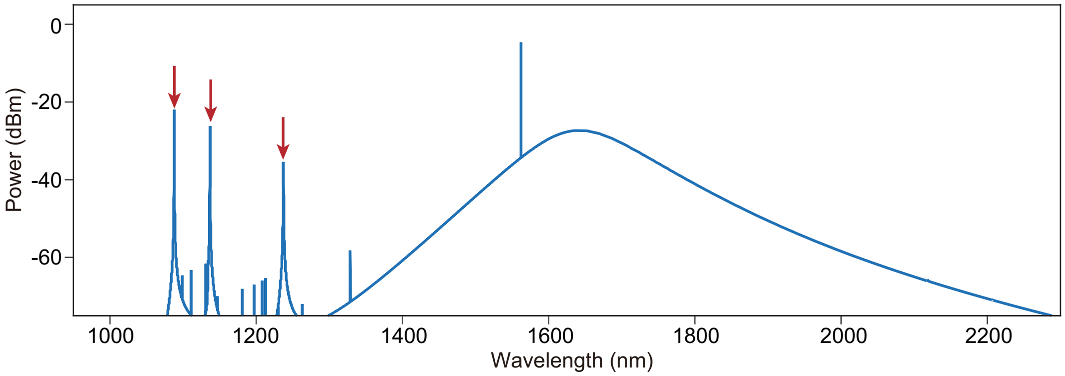

Fig. S3. **Simulated spectrum of the octave-spanning 25 GHz soliton microcomb using the complete model.** The red arrow indicates the dispersive waves arising from higher-order dispersion, whereas the other peaks result from avoided mode crossings.

III. DEVICE CHARACTERIZATION

A. Dispersion

The dispersion of the microresonators is measured by sweeping several widely tunable lasers (Toptica CTL series) across the resonances while recording the transmission signal with a photodetector. For frequency calibration, part of the laser power is split before entering the microresonator and routed through an unbalanced Mach–Zehnder interferometer (UMZI), which generates a sinusoidal reference signal. The FSR and dispersion of the UMZI in each spectral band are calibrated using a vector spectrum analyzer[14]. We also perform finite element simulations of broadband dispersion for each device using its respective geometry. Both the simulated dispersion and experimental data are shown in Fig. S4. In the plots, the integrated dispersion is defined as $D_{\mathrm{int}}(\mu) = \omega_\mu - \omega_0 - \mu D_1$, where ω_μ is the resonant frequency of the μ_{th} mode, and D_1 is the FSR in angular frequency. The integrated dispersion of optical modes shifted by nD_1 is given by $D_{\mathrm{int}}(\mu \pm n) = D_{\mathrm{int}}(\mu) \mp nD_1$, where n is an integer. The hypothetical soliton comb frequencies in the relative frequency frame are given by $\Delta\omega_{\mu,\mathrm{comb}} = \mu\omega_{\mathrm{r}} + \omega_{\mathrm{p}} - \omega_0 - D_1\mu$, where $\omega_0 - \omega_{\mathrm{p}}$ represents the pump-NR detuning and ω_{r} is the soliton repetition rate[15]. The phase-matched and quasi-phase-matched locations of dispersive waves are predicted using the conditions $D_{\mathrm{int}}(\mu) = \Delta\omega_{\mu,\mathrm{comb}}$ and $D_{\mathrm{int}}(\mu + n) = \Delta\omega_{\mu,\mathrm{comb}}$ $(n = 1, 2)$, respectively[8,16]. The predicted dispersive wave location does not match perfectly with the experiment, which can be attributed to the residual difference between the simulated and actual dispersion profiles.

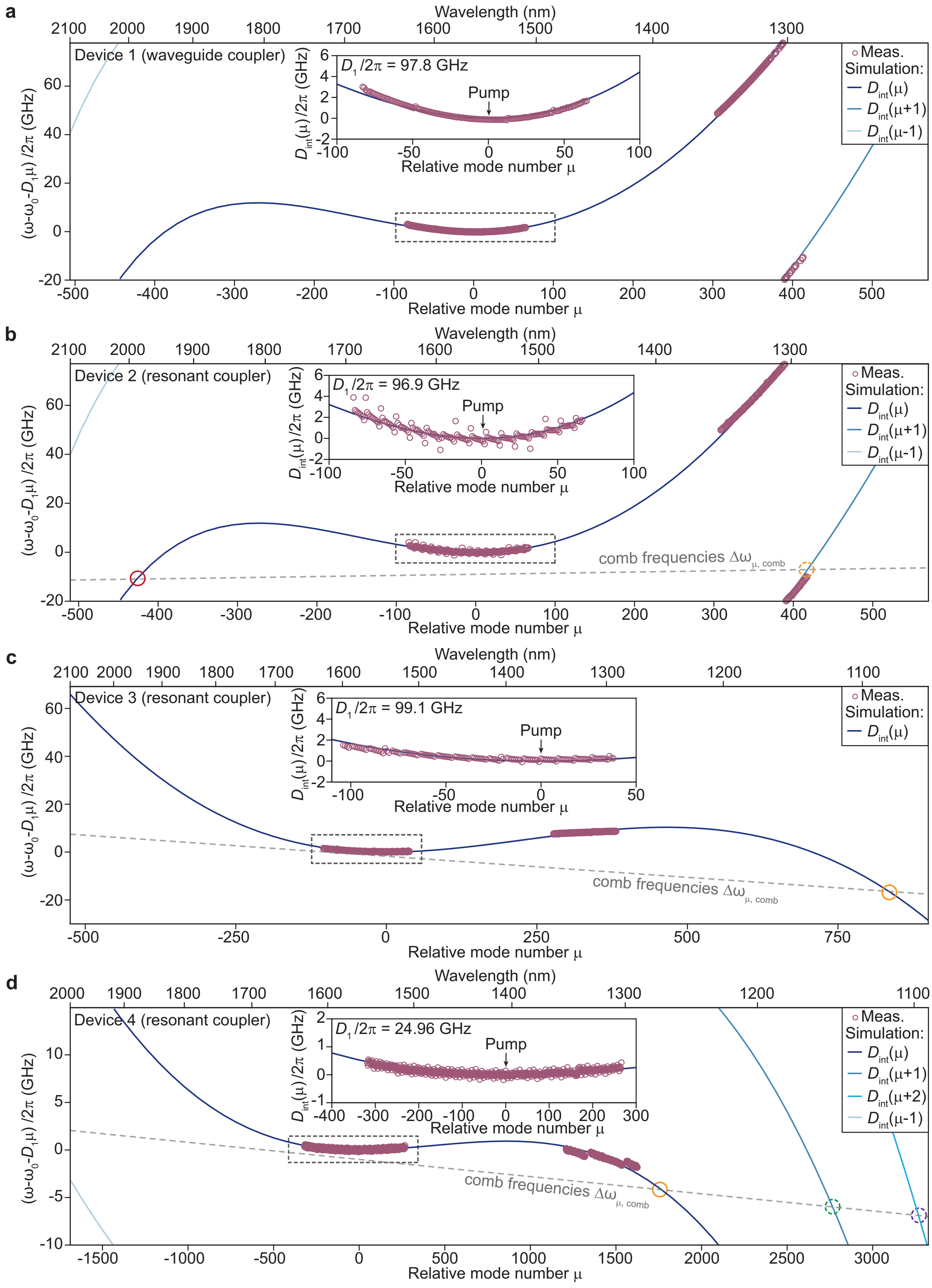

Fig. S4. **Mode family dispersion.** Integrated dispersion of the NRs in Device 1-4, respectively. Measured and simulated D_{int} values are denoted by purple circles and blue curves, respectively. $D_{\text{int}}(\mu)$ and its shifted values $D_{\text{int}}(\mu \pm n) = D_{\text{int}}(\mu) \mp nD_1$ ($n = 1, 2$) are represented in distinct shades of blue. Dashed grey lines correspond to the hypothetical soliton comb frequencies in the relative frequency frame, with their non-zero slope indicating a repetition rate offset from the NR's FSR. Circles mark dispersive wave locations: solid for phase matching $D_{\text{int}}(\mu) = \Delta\omega_{\mu,\text{comb}}$ and dashed for quasi-phase matching $D_{\text{int}}(\mu + n) = \Delta\omega_{\mu,\text{comb}}$ ($n = 1, 2$). Insets: zoom-in view of $D_{\text{int}}(\mu)$ values near the pump frequency.

B. Coupling design and experimental verification of coupled microresonators

The coupling strengths in our devices are carefully designed to balance efficient power extraction, broadband microcomb generation and low pump-power operation . For the nonlinear resonator (NR), the coupling to the bus waveguide is optimized to provide sufficient output power while maintaining a high loaded Q factor to ensure low pump-power operation. For the resonant coupler (RC), the coupling to the bus waveguide is intentionally increased to suppress unwanted parametric oscillations within the RC. The inter-resonator coupling strength is designed to provide a strong enhancement factor while minimizing nonlinear loss induced by comb generation in the NR. The pulley coupler is employed for coupling between the ring and the bus waveguide, whereas the finger-shaped NR and racetrack RC are coupled to the waveguide through their straight sections. The resonator–bus coupling is simulated following the coupled-mode formulation in Ref.[17], and the inter-resonator coupling is evaluated using the approach described in Ref.[2].

All devices are fabricated using high-yield electron-beam lithography (EBL), which ensures high fidelity to the designed geometry and stable intrinsic Q factors across the wafer[18]. To evaluate the correspondence between design and experiment, we compare the target and measured inter-resonator coupling rates and the corresponding enhancement factors calculated using Eq. S36, as shown in Fig. S5. The blue shaded region represents the variation in the enhancement factor when the NR's external coupling $Q_{\mathrm{e,NR}}$ deviates by $\pm 1 \times 10^6$ from the design value. Two representative devices from the main text are shown.

Device 2, designed for high-power ultra-broadband microcomb generation, employs a large NR dispersion and therefore requires a higher enhancement factor to sustain the large detuning range.

Device 3, designed for octave-spanning microcomb generation, has a smaller NR dispersion, for which an enhancement of several tens is sufficient for spectral broadening and its ultimate bandwidth is limited by the Raman effect and higher-order dispersion.

Measured coupling strengths agree well with design targets, confirming that the RC architecture maintains robust pump enhancement even within typical fabrication tolerances.

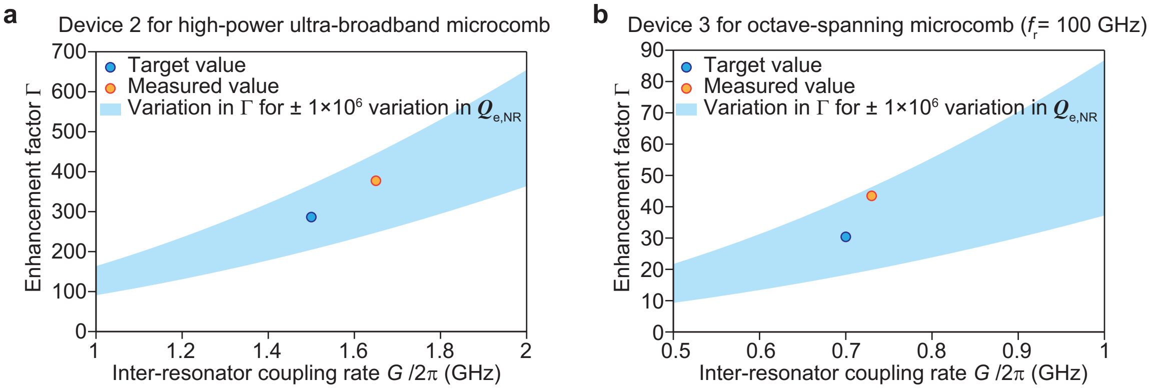

Fig. S5. **Theoretical enhancement factor (Γ) versus inter-resonator coupling rate (G) for Device 2 and 3.** Blue shaded area indicate the variation in the enhancement factor for $\pm 1 \times 10^6$ variation in $Q_{\mathrm{e,NR}}$.

IV. ADDITIONAL EXPERIMENTAL RESULTS

A. Characterization of tuning process

We employ a probe laser to visualize the tuning dynamics (Fig. S6a). Coupled from either the through port or the drop port and collected at the input, the probe signal interrogates the hybridized resonances of the NR and RC. After soliton initiation, we scan the frequency of the probe laser and observe a narrow beatnote resulting from interference with back-reflected pump (Fig. S6c-e). Broader peaks correspond to hybridized resonances, from which the RC and NR resonant frequencies are deduced (see Section. IV B).

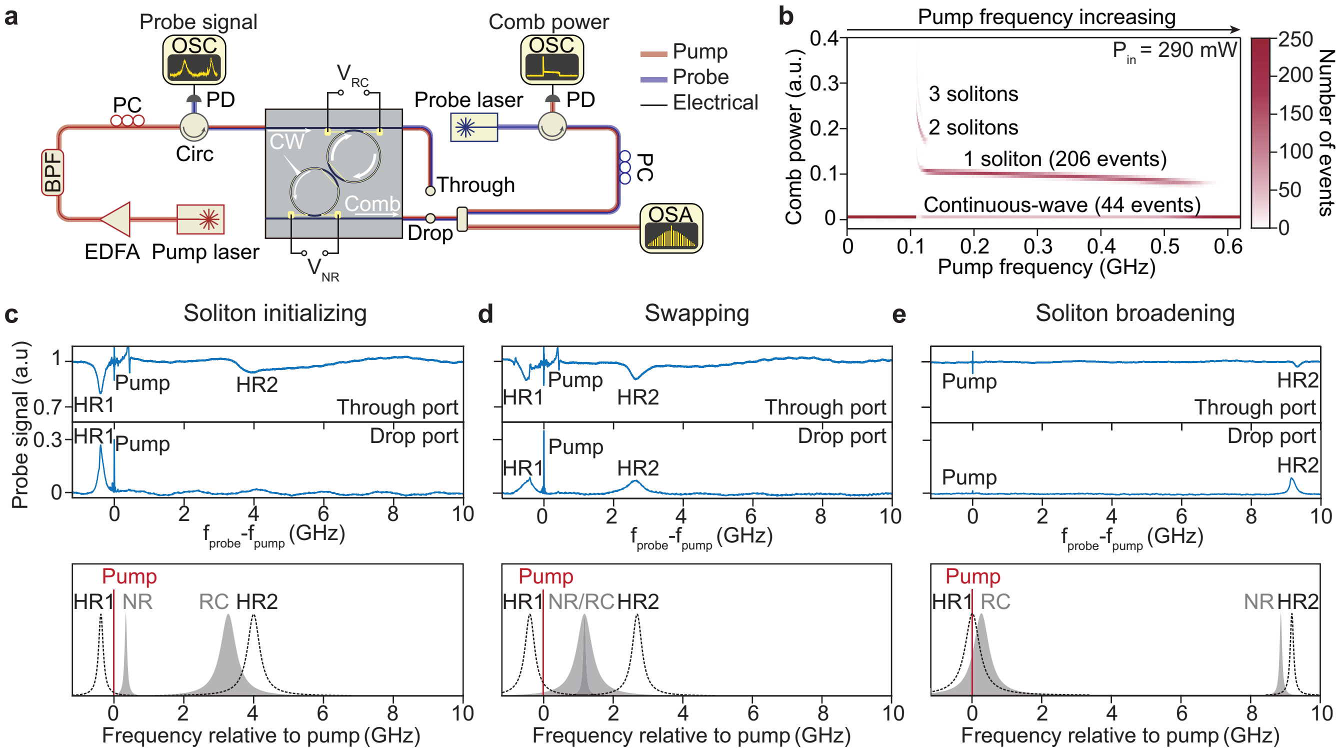

Fig. S6. **Tuning process.** **a,** Experimental setup. EDFA: erbium-doped fiber amplifier; BPF: band-pass filter; PC: polarization controller; Circ: circulator; PD: photodetector; OSC: oscilloscope; OSA: optical spectrum analyzer. **b,** Comb power versus pump laser frequency for 250 consecutive scans. The number of events is indicated by color. **c–e,** Sequential stages for generating ultra-broadband solitons in a resonantly-coupled NR. Top panel: recorded probe signals when the probe laser is launched from the through and drop ports. The hybridized resonances (HRs) and the beat note with the backreflected pump are indicated. Bottom panel: reconstructed frequencies of NR and RC relative to the pump.

B. Resonant frequencies of coupled microresonators

The hybridization of the pump resonances in the NR and RC is described by the following coupled equations:

$$\frac{\partial b_0}{\partial T} = -\frac{\kappa_{\rm RC}}{2} b_0 - i\delta\omega_{\rm RC} b_0 + iGa_0 + \sqrt{\kappa_{\rm e,RC}} s_{\rm in}, \tag{S48}$$

$$\frac{\partial a_0}{\partial T} = -\frac{\kappa_{\rm NR}}{2} a_0 - i\delta\omega_{\rm NR} a_0 + iGb_0, \tag{S49}$$

Here, $s_{\rm in} = \sqrt{P_{\rm in}/\hbar\omega_0}$, and $|s_{\rm in}|^2$ represents the photon flux of the pump. At the steady state, the intracavity field is given by:

$$b_0 = \frac{\sqrt{\kappa_{\rm e,RC}}}{i\left(\delta\omega_{\rm RC} - \frac{G^2\delta\omega_{\rm NR}}{\delta\omega_{\rm NR}^2+\kappa_{\rm NR}^2/4}\right) + \frac{\kappa_{\rm RC}}{2} + \frac{G^2\kappa_{\rm NR}/2}{\delta\omega_{\rm NR}^2+\kappa_{\rm NR}^2/4}} \cdot s_{\rm in}, \tag{S50}$$

$$a_0 = \frac{iG\sqrt{\kappa_{\rm e,RC}}}{i\left(\frac{\delta\omega_{\rm RC}\kappa_{\rm NR}+\delta\omega_{\rm NR}\kappa_{\rm RC}}{2}\right) - \delta\omega_{\rm RC}\delta\omega_{\rm NR} + \frac{\kappa_{\rm RC}\kappa_{\rm NR}}{4} + G^2} \cdot s_{\rm in}. \tag{S51}$$

According to the input-output formalism,

$$s_{\rm out,RC} = -s_{\rm in} + \sqrt{\kappa_{\rm e,RC}}b_0, \tag{S52}$$

$$s_{\rm out,NR} = \sqrt{\kappa_{\rm e,NR}}a_0, \tag{S53}$$

where $s_{\rm out,RC(NR)}$ represents the output field at the through (drop) port. Thus, the transmission spectra at the through and drop are expressed as

$$\left|\frac{s_{\rm out,RC}}{s_{\rm in}}\right|^2 = \frac{\left(\delta\omega_{\rm RC} - \frac{G^2\delta\omega_{\rm NR}}{\delta\omega_{\rm NR}^2+\kappa_{\rm NR}^2/4}\right)^2 + \left(\frac{\kappa_{\rm 0,RC}-\kappa_{\rm e,RC}}{2} + \frac{G^2\kappa_{\rm NR}/2}{\delta\omega_{\rm NR}^2+\kappa_{\rm NR}^2/4}\right)^2}{\left(\delta\omega_{\rm RC} - \frac{G^2\delta\omega_{\rm NR}}{\delta\omega_{\rm NR}^2+\kappa_{\rm NR}^2/4}\right)^2 + \left(\frac{\kappa_{\rm RC}}{2} + \frac{G^2\kappa_{\rm NR}/2}{\delta\omega_{\rm NR}^2+\kappa_{\rm NR}^2/4}\right)^2}, \tag{S54}$$

$$\left|\frac{s_{\rm out,NR}}{s_{\rm in}}\right|^2 = \frac{G^2\kappa_{\rm e,RC}\kappa_{\rm e,NR}}{\left(\frac{\delta\omega_{\rm RC}\kappa_{\rm NR}+\delta\omega_{\rm NR}\kappa_{\rm RC}}{2}\right)^2 + \left(\delta\omega_{\rm RC}\delta\omega_{\rm NR} - \frac{\kappa_{\rm RC}\kappa_{\rm NR}}{4} - G^2\right)^2}. \tag{S55}$$

Based on the above equations, the calculated resonant frequencies of RC and NR relative to the pump for the three stages presented in Fig. 2d-f and Fig. S6c-e are: $\delta\omega_{\rm NR}/2\pi = 0.34$ GHz, $\delta\omega_{\rm RC}/2\pi = 3.27$ GHz for soliton initializing, $\delta\omega_{\rm NR}/2\pi = 1.24$ GHz, $\delta\omega_{\rm RC}/2\pi = 1.24$ GHz for swapping, and $\delta\omega_{\rm NR}/2\pi = 8.9$ GHz, $\delta\omega_{\rm RC}/2\pi = 0.3$ GHz for soliton broadening.

C. Optical spectrum

Figure S7 presents the optical spectra measured at both the drop and through ports while the NR operates in the high-power ultra-broadband soliton state, under a pump power of 290 mW applied to the bus waveguide. Notably, the spectrum obtained from the through port exhibits high-power teeth, which is attributed to mode crossings induced by the vernier effect between the resonances of NR and RC[19].

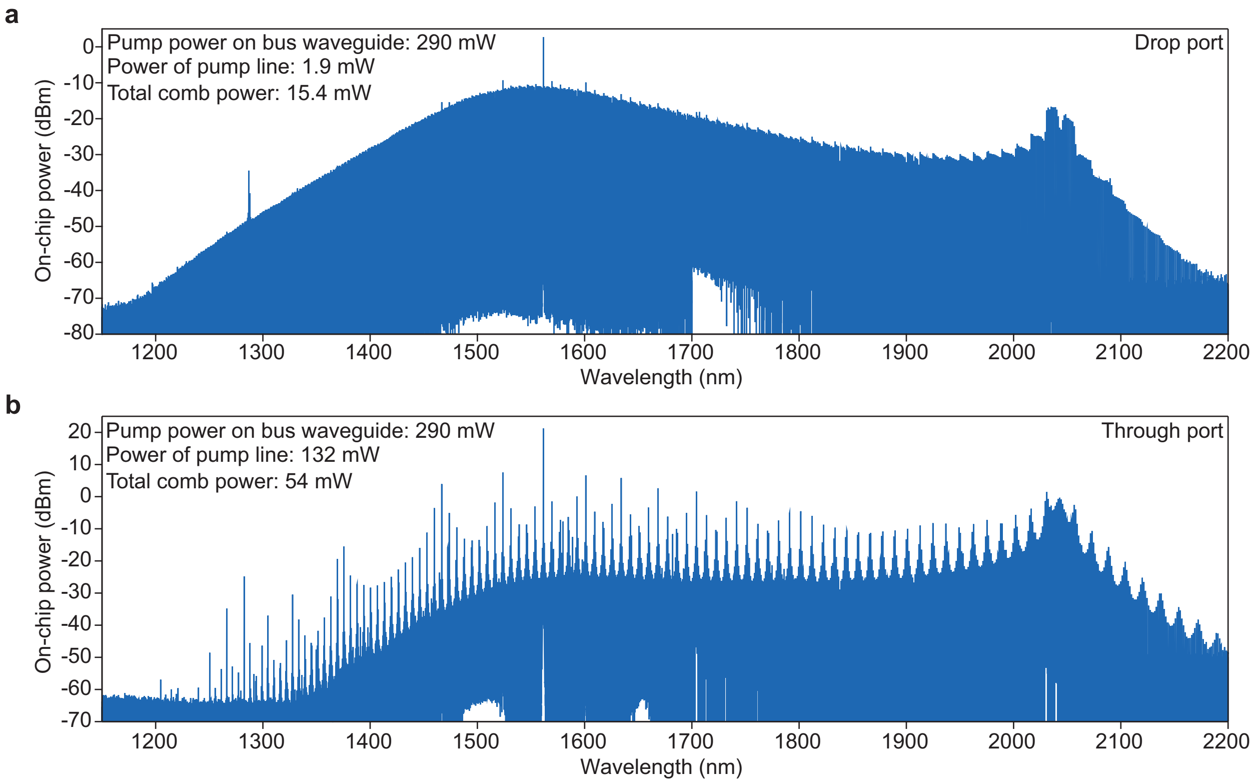

Fig. S7. **Optical spectra of the high-power ultra-broadband soliton microcomb. a,** Spectrum from the drop port. **b,** Spectrum from the through port.

D. Autocorrelation

The temporal profile of the high-power ultra-broadband soliton microcomb is characterized using an autocorrelator (APE pulseCheck), where the dispersion is compensated using a dispersion-compensating fiber. Fitting of the autocorrelation trace reveals that the soliton pulse exhibits a full-width-at-half-maximum (FWHM) of 42.7 fs (Fig. S8). It should be noted that the measured FWHM exceeds the 15.84 fs FWHM inferred from the optical spectrum (Fig. S7a), likely due to the limited bandwidth offered by the frequency-doubling crystal in the autocorrelator.

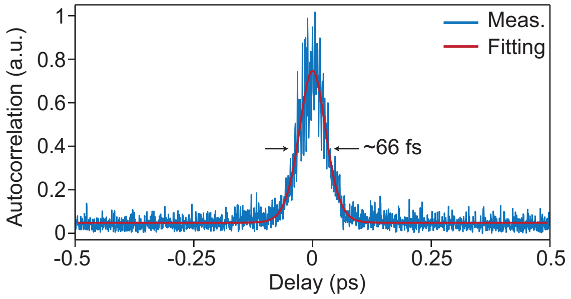

Fig. S8. **Intensity autocorrelation of the high-power ultra-broadband soliton microcomb.** The full-width-at-half-maximum of the autocorrelation trace is 66 fs.

E. Measurement of the repetition rate

The repetition rate of the high-power ultra-broadband soliton microcomb is determined via electro-optic (EO) downconversion[20] (Fig. S9a). In this measurement, two adjacent comb lines are isolated using a band-pass filter and

subsequently modulated with a phase modulator operating at $f_{\mathrm{RF}} = 40$ GHz. This modulation generates sidebands around each comb line, and a pair of sidebands is selected by an additional band-pass filter. The resulting low-frequency beat note, f_{beat}, is detected by a high-speed photodetector. By measuring the beat note using an electrical spectral analyzer, we can deduce the comb spacing, which is given by

$$f_{\mathrm{rep}} = 2f_{\mathrm{RF}} + f_{\mathrm{beat}}. \tag{S56}$$

In our experiments, $f_{\mathrm{beat}} = 16.888$ GHz, indicating a soliton microcomb repetition rate of $f_{\mathrm{rep}} = 96.888$ GHz. The phase noise of the downconverted beat note, $S_{\phi,\mathrm{beat}}$, is related to the phase noise of the repetition rate, $S_{\phi,\mathrm{rep}}$, and that of the RF source, $S_{\phi,\mathrm{RF}}$, according to

$$S_{\phi,\mathrm{beat}} = S_{\phi,\mathrm{rep}} + 4S_{\phi,\mathrm{RF}}. \tag{S57}$$

Figure S9b presents the measured phase noise of the downconverted beatnote using a phase noise analyzer. For offset frequencies below 10 kHz, $S_{\phi,\mathrm{beat}}$ predominantly reflects $S_{\phi,\mathrm{rep}}$, as $S_{\phi,\mathrm{RF}}$ is comparatively negligible. At offset frequencies above 30 kHz, the noise is primarily limited by the RF source. These findings demonstrate the mutual coherence of the generated ultra-broadband soliton microcomb.

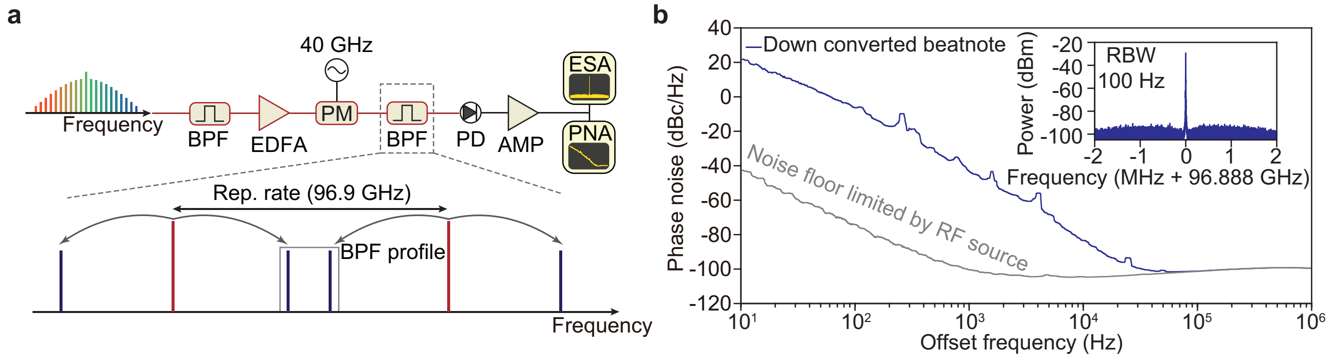

Fig. S9. **Coherence of the high-power ultra-broadband soliton microcomb. a,** Experimental setup for electro-optic (EO) downconversion. BPF: band-pass filter; EDFA: erbium-doped fiber amplifier; PM: phase modulator; PD: photodetector; AMP: electrical amplifier; ESA: electrical spectral analyzer; PNA: phase noise analyzer. The lower schematic illustrates the phase-modulated microcomb spectra entering the BPF, where EO-modulated sidebands (blue) of the adjacent microcomb modes (red) overlap to produce the downconverted beat note. **b,** Single-sideband phase noise of the downconverted beat note, along with the RF source contribution. Inset: beat note corresponding to the microcomb repetition rate. RBW: resolution bandwidth.

F. Coherence of the dispersive wave

To assess the coherence of the dispersive wave of the 100 GHz repetition-rate octave-spanning soliton microcomb, we perform heterodyne beat note measurements. The soliton microcomb is combined with a tunable CW laser (Toptica CTL series) using a 90:10 fiber coupler. The combined signal is then detected by a silicon photodetector (New Focus Model 1801), which is responsive only to wavelengths below 1050 nm. In this configuration, only the dispersive wave components fall within the detector's bandwidth. The resulting beat notes exhibit a narrow linewidth, confirming the coherence of the dispersive wave (Fig. S10).

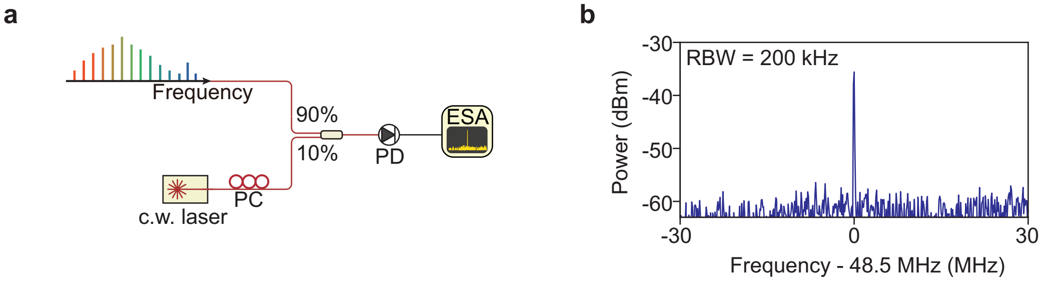

Fig. S10. **Heterodyne beat note measurements of the dispersive wave. a,** Experimental setup. PC: polarization controller. **b,** The measured beat note of the 100 GHz repetition-rate octave-spanning soliton microcomb with a narrow linewidth laser positioned at 1011.2 nm.

G. Setup for coherence characterization of octave-spanning soliton microcombs.

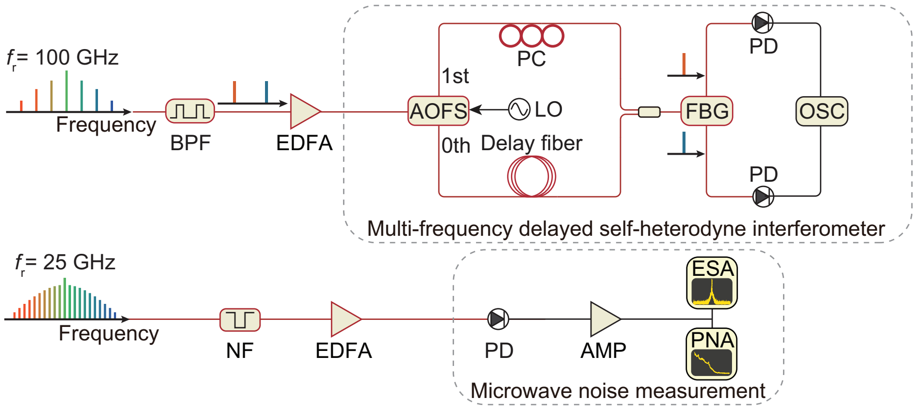

Fig. S11. **Experimental setup for coherence characterization of octave-spanning soliton microcombs.** BPF: band-pass filter; EDFA: erbium-doped fiber amplifier; AOFS: acousto-optic frequency shifters. PD: photodetector; LO: local oscillator; FBG: fiber Bragg grating; OSC: oscilloscope; NF: notch filter; AMP: electrical amplifier; ESA: electrical spectral analyzer; PNA: phase noise analyzer.

[1] Lugiato, L. A. & Lefever, R. Spatial dissipative structures in passive optical systems. *Physical Review Letters* **58**, 2209–2221 (1987).

[2] Yuan, Z. *et al.* Soliton pulse pairs at multiple colours in normal dispersion microresonators. *Nature Photonics* **17**, 977–983 (2023).

[3] Bao, C. *et al.* Nonlinear conversion efficiency in Kerr frequency comb generation. *Optics Letters* **39**, 6126–6129 (2014).

[4] Herr, T. *et al.* Temporal solitons in optical microresonators. *Nature Photonics* **8**, 145–152 (2014).

[5] Godey, C., Balakireva, I. V., Coillet, A. & Chembo, Y. K. Stability analysis of the spatiotemporal Lugiato-Lefever model for Kerr optical frequency combs in the anomalous and normal dispersion regimes. *Physical Review A* **89**, 063814 (2014).

[6] Parra-Rivas, P., Gomila, D., Matías, M. A., Coen, S. & Gelens, L. Dynamics of localized and patterned structures in the Lugiato-Lefever equation determine the stability and shape of optical frequency combs. *Physical Review A* **89**, 043813 (2014).

[7] Yi, X., Yang, Q.-F., Yang, K. Y., Suh, M.-G. & Vahala, K. Soliton frequency comb at microwave rates in a high-Q silica microresonator. *Optica* **2**, 1078–1085 (2015).

[8] Brasch, V. *et al.* Photonic chip–based optical frequency comb using soliton Cherenkov radiation. *Science* **351**, 357–360 (2016).

[9] Nielsen, A. U. *et al.* Nonlinear localization of dissipative modulation instability. *Physical Review Letters* **127**, 123901 (2021).

[10] Carmon, T., Yang, L. & Vahala, K. J. Dynamical thermal behavior and thermal self-stability of microcavities. *Optics Express* **12**, 4742–4750 (2004).

[11] Parra-Rivas, P., Gomila, D., Leo, F., Coen, S. & Gelens, L. Third-order chromatic dispersion stabilizes Kerr frequency combs. *Optics Letters* **39**, 2971–2974 (2014).

[12] Li, Z. *et al.* Observations of existence and instability dynamics of near-zero-dispersion temporal Kerr cavity solitons. *Physical Review Research* **3**, 043207 (2021).

[13] Helgason, Ó. B. *et al.* Surpassing the nonlinear conversion efficiency of soliton microcombs. *Nature Photonics* 1–8 (2023).

[14] Luo, Y.-H. *et al.* A wideband, high-resolution vector spectrum analyzer for integrated photonics. *Light: Science & Application* **13**, 83 (2024).

[15] Yi, X. *et al.* Single-mode dispersive waves and soliton microcomb dynamics. *Nature Communications* **8**, 14869 (2017).

[16] Anderson, M. H. *et al.* Zero dispersion Kerr solitons in optical microresonators. *Nature Communications* **13**, 4764 (2022).

[17] Chuang, S.-L. A coupled mode formulation by reciprocity and a variational principle. *Journal of Lightwave Technology* **5**, 5–15 (1987).

[18] Wang, Y. *et al.* Compact turnkey soliton microcombs at microwave rates via wafer-scale fabrication. *arXiv preprint arXiv:2502.10941* (2025).

[19] Hu, Y. *et al.* High-efficiency and broadband on-chip electro-optic frequency comb generators. *Nature Photonics* **16**, 679–685 (2022).

[20] Del'Haye, P., Papp, S. B. & Diddams, S. A. Hybrid electro-optically modulated microcombs. *Physical Review Letters* **109**, 263901 (2012).
